# Validation of the Constitution in Chinese Medicine Questionnaire: Does the Traditional Chinese Medicine Concept of Body Constitution Exist?

**DOI:** 10.1155/2013/481491

**Published:** 2013-04-24

**Authors:** Wendy Wong, Cindy Lo Kuen Lam, Vivian Taam Wong, Zhi Min Yang, Eric T. C. Ziea, Andrew Ka Lun Kwan

**Affiliations:** ^1^Department of Family Medicine and Primary Care, the University of Hong Kong, Hong Kong; ^2^Chinese Medicine Department and Integrative Medicine, the Hong Kong Hospital Authority, Hong Kong; ^3^Affiliated Hospital of Guangzhou University of TCM, Hong Kong

## Abstract

The study aims to adapt and validate the Constitution in Chinese Medicine Questionnaire (CCMQ) in Hong Kong Chinese people. 10 patients and 10 Chinese medicine practitioners (CMP) confirmed the content validity (CVI: 50%–100%) of CCMQ. 1084 HK subjects completed a cross-sectional study with 98.6% who could be classified into one or more BC types. Scaling success rates were 85.7%–100% for the 9 BC scales. Construct validity was supported by moderate correlations between CCMQ and SF-12v2 scores. The confirmatory factor analysis showed a reproducible structure as hypothesized. People with gentleness BC type had better health-related quality of life, HRQOL, than those with other (imbalanced) BC types. Internal consistency (reliability) (Cronbach's alpha  >  0.6) and test-retest reliability were also satisfactory (ICC > 0.6) for all scales. However, the sensitivity and specificity in predicting the BC types diagnosed by CMP were only fair, ranging from 42.7% to 82.7%. 27.6% of subjects had a change from the imbalanced BC types to gentleness BC type after 6 months. The CCMQ was adapted for HK Chinese people and proved to be valid, reliable, and responsive. People classified to have imbalanced BC types had significantly lower HRQOL than gentleness BC type, which supported the validity and importance of the TCM concept of the physiological BC type.

## 1. Introduction

Body constitution (BC), an ancient core concept in traditional Chinese medicine (TCM), is widely applied in daily practice by Chinese medicine practitioners (CMP), but there is little standardization on its measurement. Many debate and challenge on this which long regarded as subjective. Studies have found low agreement on the BC type diagnoses among CMP [1–3], which prevents proper selection of subjects for clinical trials and hinders the development of TCM research [1–3]. Enhancing the consistencies of the fundamental classification of physiological BC type under TCM diagnoses has been a major development direction since the 1990s [4]. With the help of objective and standardized questions, BC concept can be helpful for further investigation. To improve the consistency of diagnosis of BC type, TCM scholars have developed structured questionnaire to classify BC type [4, 5]. The most common BC instruments are the Constitution in Chinese Medicine Questionnaire (CCMQ) developed Wang et al. in Mainland China for measuring BC type [6–9] and the Body Constitutions Questionnaire (BCQ) developed by Su et al. in Taiwan [10–19]. There are some data supporting the face validity by expert panel discussion, validity (construct validity and criterion validity), and reliability (internal consistency) on 2854 subjects in Mainland China of these questionnaires in populations of their origins [7–9]; they have never been evaluated for applicability and validity in other Chinese populations.

The Constitution in Chinese Medicine Questionnaire (CCMQ) was developed by Wang et al. in Mainland China [6], by consensus among experts in TCM BC types. It has 60 items measuring the 9 BC types: gentleness, Qi-deficiency, Yang-deficiency, Yin-deficiency, phlegm-wetness, wetness-heat, blood-stasis, Qi-depression, and special diathesis. It was pilot-tested in the Beijing population to establish its face validity. Its reliability and construct validity were proven in 2500 people from five different geographical districts in China [20]. Although the CCMQ has been used in China nationwide campaigns since 2008 [21] mainly in epidemiological studies on the prevalence of BC types [22], it has never been tested or used on Chinese populations outside Mainland China including that of Hong Kong where the lifestyle, linguistic, health believes, and culture might be different [23]. The content and construct validity and other psychometric properties of CCMQ need to be confirmed before it could be applied to Chinese populations in Hong Kong or overseas. Confirmation of this would support the application of the CCMQ in cross-region and cross-country research on BC types. In addition, this is the first study to provide empirical data to validate the ancient concept in TCM theory which is important to future TCM clinical trials or investigation.

### 1.1. Aims

The aim of this study was to adapt and validate the CCMQ in Hong Kong Chinese in order to establish evidence on its content and construct validity, reliability, sensitivity, and responsiveness.

### 1.2. Objectives

(1) To adapt the CCMQ to a HK version that is linguistically valid for Cantonese speaking Chinese in Hong Kong.
(2) To evaluate the content validity of the HK version of the CCMQ by Chinese medicine practitioners (CMPs) experts and lay persons in Hong Kong.
(3) To test the psychometric properties including construct validity by scaling assumptions, factor structure and known group comparison, criterion validity, reliability, sensitivity, and responsiveness of the HK version of CCMQ.

## 2. Methods

### 2.1. Subjects

To evaluate the content validity of CCMQ, convenient samples of 10 patients and 10 Chinese medicine practitioners (CMP), respectively, were recruited from June to July, 2010, to complete the CCMQ and cognitive debriefings. A convenient age-gender stratified sample of Cantonese speaking patients was recruited from the Ap Lei Chau General outpatient clinic (ALCGOPC), and all subjects completed a written consent form. All CMP were academically qualified with a bachelor's degree in CM and more than 5 years of clinical experience (average 7.2–8.4 years). The characteristics of the subjects are shown in Table 1.

### 2.2. Data Collection

2128 eligible patients attending a Western medicine (WM) outpatient clinic (ALCGOPC) and two Chinese medicine outpatient clinics were invited and 1084 patients participated in the cross-sectional validation study from July to October, 2010. The characteristics of the subjects are summarized in Table 1.

The CCMQ was reviewed and adapted to be linguistically appropriate for Chinese people in Hong Kong by a professional translator to form a draft HK version of the CCMQ. This was sent to 10 Chinese medicine practitioners (CMPs) with the completion of the cognitive debriefing questionnaire (see Supplementary Appendix A in Supplementary Material available online at http://dx.doi.org/10.1155/2013/481491). 10 lay subjects (patients from an ALCGOPC) completed the cognitive debriefing questionnaire administered by a trained interviewer whose responses to the open questions were recorded in verbatim. The standard procedure and questions of cognitive debriefing were followed [23, 24]. 

To investigate the construct validity, reliability, sensitivity, and responsiveness of the CCMQ, each of the 1068 patients ≥18 years old answered the Hong Kong version of the CCMQ, the HK Chinese Short Form-12 version 2 Health Survey (SF-12v2) and a structured questionnaire on sociodemographics and chronic morbidity (Supplementary Appendix B) before assessment by a Chinese medicine practitioner (CMP). The CMP, blinded on the CCMQ results, assessed the subject and completed a structured evaluation form to indicate the BC types and severity (Supplementary Appendix C). 

To evaluate test-retest reliability, 225 patients attending the ALCGOPC for routine chronic disease followup were retested with the CCMQ (HK version) administered by telephone 2 weeks after the first test.

**Figure 1:**
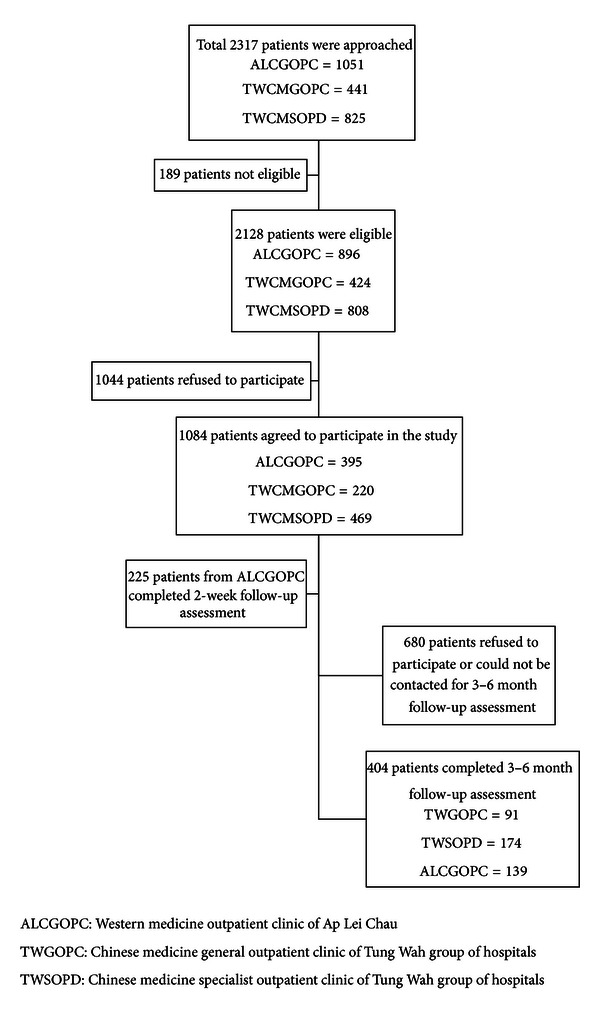
Patient recruitment.

1084 subjects agreed to a follow-up survey in 3 to 6 months, and 404 subjects completed the telephone interviews with the HK versions of the CCMQ and SF-12v2 in addition to a one-item Global Rating Scale (GRS) on change in health condition [25] to access the responsiveness of the CCMQ (HK version). The recruitment of patients was shown in Figure 1.

### 2.3. Sample Size Calculation

The sample size for cognitive debriefing study on content validity was recommended by an international group [24]. The sample size for construct validity and reliability study was powered to determine the proportion of subjects with each BC type based on the most conservative estimate of 50%. In order to restrict the width of the 95% confidence interval of this proportion estimated to a two-sided standard error of 3%, a sample size of 1068 was needed. The sample size was also sufficient for the psychometric testing of the CCMQ based on the general guideline of least 7 subjects for each item [26].

### 2.4. Study Instruments and Outcome Measures

#### 2.4.1. Constitution in Chinese Medicine Questionnaire (CCMQ)

The Constitution in Chinese Medicine Questionnaire (CCMQ) [6, 7, 9, 27] consists of 60 items to classify a person into one or more of nine BC types: gentleness (8 Items), Qi-deficiency (8 Items), Yang-deficiency (7 Items), Yin-deficiency (8 Items), phlegm-wetness (8 Items), wetness-heat (6 Items), blood-stasis (7 Items), Qi-depression (7 Items), and special diathesis (7 Items). Coexistence of multiple imbalanced BC types was possible which is consistent with the TCM theories. The scoring algorithm proposed in the original CCMQ was adopted in this study. A higher score in the CCMQ BC scale indicates a higher likelihood of the specific BC type, and a score of 30 is set as threshold for case definition.

#### 2.4.2. The Chinese (HK) SF-12v2 Health Survey

The Chinese (HK) Short Form-12 version 2 (SF-12v2) Health Survey is a health-related quality of life (HRQOL) measure that has been translated, validated, and normed on the general Chinese population in Hong Kong [28]. It consists of 12 items measuring eight HRQOL domains on physical functioning, physical role, bodily pain, general health, vitality, social functioning, emotional role, and mental health. The scale scores can be summarized into two (physical and mental) summary scores. Higher scores indicate better HRQOL.

#### 2.4.3. The Global Rating on Change Scale (GRS) on Change in Health

The Global Rating on Change Scale (GRS) asked the subjects to rate on the change in his/her own illness condition since the initial TCM/WM consultations. The response was given as a score of zero for no change, +1, 2, or 3 for different degrees of improvement, and −1, 2, or 3 for different degrees of deterioration.

## 3. Data Analysis

### 3.1. Content Validity

The content validity indexes (CVI), the proportion of subjects who gave a positive rating, on clarity and relevance, was calculated for each item [29]. A CVI ≥ 80% was considered satisfactory [29]. The Chinese medicine practitioners (CMPs) and lay subjects' answers to the open-ended cognitive debriefing questions were reviewed by an expert panel that consisted of the original author of the measure and experts in Health-related quality of life (HRQOL) research. Discrepancies between subjects' interpretation of the meaning of the items or response options and the intended meaning were highlighted, and items that were found to be unclear were identified. Revisions were made to the problematic item(s), taking into account respondents' suggested rewording, to form the final HK version of the CCMQ. 

### 3.2. Construct Validity

#### 3.2.1. Scaling Assumptions

The scaling assumptions were tested by (i) item-scale correlations, against the hypothesis that there should be substantial linear correlations (*r* ≥ 0.4); (ii) scaling success, defined as the item and hypothesized-scale correlation being greater than item and competing-scale correlations. This proportion of total number of item-scale correlations of all items in each scale that were successful was calculated [30]. Floor and ceiling effects of CCMQ scales were considered significant if over 15% of subjects got a minimal or maximum baseline score for each question [31].

#### 3.2.2. Confirmatory Factor Analysis (CFA)

CFA was used to determine whether the items load onto the hypothesized subscales by the Satorra-Bentler scaled chi-square statistic [32]. The Confirmatory factor analysis (CFA) models were considered to have acceptable model fit if root mean square error of approximation (RMSEA) values, its 90% confidence interval, standardized root mean square residual (SRMR), were close to 0.08 or below, and comparative fit index (CFI), Tucker-Lewis index (TLI), and incremental fit index (IFI) values were close to 0.95 or greater [33].

#### 3.2.3. Known-Group Comparison

Correlations between scores of corresponding subscales of CCMQ were calculated by Spearman's correlation. Known-group comparison would be considered by studying the difference of CCMQ and SF-12 scores by gender and age groups in Mann-Whitney *U* test and Kruskal Wallis *H* test respectively. Moreover, the SF-12v2 scores of different BC types classified by CCMQ were compared by independent samples *t*-test. It was hypothesized that subjects of the gentleness BC type of CCMQ should have the highest SF-12v2 scores because they were supposedly the most healthy.

#### 3.2.4. Criterion Validity

CMP diagnosis of the BC type was used as the “gold standard”. The sensitivity and specificity of the CCMQ in predicting the CMP BC type diagnosis were calculated. The agreement between the diagnoses by the CCMQ with the CMP was assessed by the Kappa coefficient of which “1” indicates complete agreement and “0” complete disagreement [34]. 

#### 3.2.5. Sensitivity

The sensitivity of the CCMQ (HK version) was tested by patients with different levels of demographic groups (i.e., age and genders). It was hypothesized that patients who were older or female would have higher CCMQ scores [22]. The difference between two groups was tested by Mann-Whitney *U* test, while more than two groups were tested by Kruskal-Wallis *H* test for, and *P* values less than 0.05 were considered statistically significant.

### 3.3. Reliability

Internal consistency of CCMQ was measured by Cronbach's alpha that indicates the extent to which items in a scale are homogeneous in supporting the same concept. Test-rest reliabilities of CCMQ scales were evaluated by intraclass correlations (ICC) and paired *t*-tests of the difference between 2-week test-retest scores, which were evaluated by the stability of the reproducibility of the instrument. Reliability coefficients ≥0.7 are the usual standard for group comparisons [35, 36]. 

### 3.4. Responsiveness

The proportion of subjects who had a change in the BC type classified by the CCMQ in 3–6 months was used as a measure of the responsiveness of the instruments to detect a change over time from summer to winter seasons. According to the Chinese medicine theory, 10–20% of patients would expect to have a change in their BC types. Subjects were divided into the gentleness BC type or any imbalanced BC types at the baseline survey, and the proportion of each with a change in the gentleness or imbalanced BC types classification was determined. The change in the mean scale scores of subjects who have reported a change in the health condition measured by the GRS was analyzed and this would be tested by paired *t*-test and McNemar-Bowker test.

Confirmatory factor analysis was adopted in LISREL 8.80, and other data analysis was carried out in SPSS for window 17.0. Statistical significant levels were set at *P* values of 0.05.

## 4. Results

### 4.1. Content Validity

Time taken to complete the CCMQ by patients was 10.9 ± 5.4 minutes, while it was 15.6 ± 8.9 for Chinese medicine practitioners (CMPs). The CCMQ had satisfactory CVIs (≥80%) on clarity, consistency of response options, and relevance with health in all items except for 6 items (Table 2). Three items had low rating from CMP and 3 had low rating from lay subjects. Firstly, CMP opinioned that the response options of “usually” (*Jing Chang*) and “often” (*Chang Chang*) were nondifferential, CVI of 50%. Items 3 “will you easily feeling shortage of breath (gasping for breath)” and 10 “do you think you are sentimental or fragile emotionally?” were rated as unclear by some CMP with CVIs of 60%. Some lay persons found the items of “are you capable to adapt to the external changes of the natural and social environment?,” “will you easily feel palpitation?,” and “will you feel wetness in your scrotum (men only)” unclear with CVIs of 50–60%. Although these items were thought by some subjects to be unclear, the interpretations given from either CMP or lay persons were consistent with the intended meaning of the original CCMQ.

On the other hand, the interpretation of 5 items including the response options “often” (*Chang Chang*), “shortness of breath” (*Qi Duan*), “excessive sweating” (*Xu Han*), “urticarial” (*Xun Ma Zhen*), and “purpura” (*Zi Dian*) were interpreted wrongly by some lay persons. The expert panel revised these 5 items based on the suggestions on rewording from the CMP and lay persons (Table 3) to form the HK version of the CCMQ (Supplementary Appendix D) that was used in the further validation study.

### 4.2. CCMQ Score and BC Type Distribution

The baseline score distribution, floor and ceiling effect proportions of the CCMQ, and SF-12v2 scales are shown in Table 1. No significant flooring and ceiling effect was found in CCMQ. Higher CCMQ scores indicate more severe imbalance (i.e., poor health), except for the gentleness scale. Among 1084 patients, 98.6% of subjects could be classified into at least one BC type by the CCMQ with 20% classified as the gentleness BC type (Table 4) and 64.9% had more than one imbalanced BC type. 

### 4.3. Construct Validity

Table 5 shows satisfactory item-scale correlations and scaling success rates of CCMQ scales. 

Item 53 “are you capable to adapt to the external changes of the natural and social environment?” of the gentleness scale had an item-scale correlation <0.4. 

### 4.4. CFA

Confirmatory factor analysis (CFA) (Table 6) confirmed the 9-factor structure of CCMQ as originally hypothesized. The overall fit indexes (RMSRA, SRMR, CFI, TLI, and IFI) were up to standards, which indicate that the model fit of CCMQ was good. 

### 4.5. Known Group Comparison

The correlations between the scale scores of the CCMQ and SF-12v2 are shown in Table 7. As hypothesized, there was a positive correlation between the CCMQ gentleness BC type and SF-12v2 scores; higher scores of both indicate better conditions. Significant negative correlations were found between SF-12v2 and all other CCMQ scale scores indicating worse HRQOL associated with more severe imbalanced BC types, which supported the concurrent validity of the CCMQ. The scale scores of CCMQ correlated most strongly with the VT, RE, MH, and MCS of SF-12v2.  Table 8 shows that subjects who had gentleness BC type had the highest SF-12v2 scores in all domains which further supporting the construct validity of CCMQ by known group comparison.

### 4.6. Criterion Validity

CMP diagnoses were used as a gold standard to assess the accuracy of the BC type classification by the CCMQ.  Table 11 shows that the sensitivities of the CCMQ in predicting the CMP BC type diagnosis ranged from 42.9% to 75%. The best sensitivity (75%) was for the detection of the special diathesis BC type. The specificities of the CCMQ ranged from 42.7% to 82.7% with the best (82.7%) in excluding the gentleness BC type.

### 4.7. Sensitivity

Table 12 shows the CCMQ and SF-12v2 scores classified by gender and age groups, respectively. It was found that female patients had lower mean CCMQ scores except for gentleness and wetness-heat of the CCMQ scales. Elderly subjects classified as gentleness BC type had a higher mean score than younger counterparts, but elderly subjects classified with imbalanced BC types had lower mean CCMQ scores than younger people. This indicates that the CCMQ was sensitive in detecting the differences between groups.

### 4.8. Reliability

The internal reliability of Cronbach's alpha and intraclass correlation were all satisfactory (>0.6) (Table 9). The intraclass correlation (ICC) coefficients of CCMQ ranged from 0.71 to 0.88, supporting good test-retest reliability of the scale scores.  Table 10 shows the number and proportion of people who were classified into the same BC type after 2 weeks with kappa statistics ranging from 0.318 to 0.531, which indicated poor reproducibility. 

### 4.9. Responsiveness

Table 13 shows significant difference in the mean CCMQ scale scores after 3–6 months. A significant proportion of people had a change in the classification by each BC type. 20.6% of subjects had a change from the gentleness BC type to one or more imbalanced BC types. Apart from changes in CCMQ scores, 27.6% of subjects, with any imbalanced BC types had changed to the Gentleness BC type, had reported a better global rating scale of health status (i.e., 36.6% unchanged while 6.1% worse than baseline). These results confirmed that the CCMQ was responsive to change in BC type over time either by score change or patients' reported outcome.

## 5. Discussions

### 5.1. Content Validity

The results confirmed the need to evaluate the validity of a psychometric measure before its application to a different population. Some items of the CCMQ were not clear to Chinese people in Hong Kong even though the instrument was developed in Chinese. Some concepts such as “shortness of breath” (*Qi Duan*), “urticaria” (*Feng Zhen*), or “excessive sweating” (*Xu Han*) from Chinese medicine were not well understood by Chinese in Hong Kong, as found in a previous study [23]. Western medicine dominates the health-care system in Hong Kong making people unfamiliar with these terms used only by CMP. Psychometric testing supported the construct validity, reliability, sensitivity, and responsiveness of the CCMQ scales, but the accuracy and reproducibility in BC type classifications were uncertain. 

### 5.2. BC Type Classification

The CCMQ was able to classify 98.6% subjects into at least one type of BC supporting its feasibility and acceptability. Only around 20% subjects were classified to have gentleness BC type, which was lower than the 32.1% found in population studies in China [22] probably because the study subjects were recruited from a patient population. A very high (65%) proportion of subjects had more than one BC type, which was compatible with the theory of traditional Chinese medicine and clinical experience. The identification of overlapping BC types has important implication for individualized health promotion practices in that some treatments intended to improve one BC type may be contraindicated for another BC type. However, this might have caused the low agreement between the CCMQ classification and CMP diagnoses. The CMP would usually diagnose one or at most two (a major and a minor) BC types in any one person, taking the person as a whole, but a survey instrument cannot do so. More research should be carried out on the cause, interpretation and significance of overlapping BC types. 

### 5.3. Psychometric Properties of the CCMQ

There was no floor or ceiling effect in the CCMQ scale suggesting that the instrument could be useful in monitoring improvement or deterioration in a particular BC type over time or in response to interventions [31]. 

### 5.4. Validity of the CCMQ

There were positive correlations between gentleness BC type with SF-12v2 scores but reverse correlations between the other imbalanced BC types. People with gentleness BC type are considered the healthiest; therefore, they had the highest SF-12 scores. The results were good evidence on not only the concurrent validity of the CCMQ but also the importance of imbalanced BC types and the concept of “Not Yet Ill.” 

Using the pragmatic diagnosis by CMP as the gold standard, the sensitivity and specificity of the CCMQ in predicting the BC type were lower than expected. A previous study also showed similar results [37], the authors pointed out the problem of subjectivity in the diagnosis by CMP. In our field testing of this study, we found that agreement on the BC type diagnosis between two CMP was only 52%, which was improved to 78% after standardization on the diagnostic criteria. It would be interesting to find out whether the CCMQ could be used as an instrument to standardize BC type diagnosis among CMP. The cut-off score of 30 for a positive BC type might need to be recalibrated to improve its predictive accuracy. 

### 5.5. Reliability

The reliability coefficients of the CCMQ scales were comparable to the established SF-12v2 Health Survey, meaning that the CCMQ scales were consistent and reproducible. However, there was relatively poor reproducibility in the classification of the same BC type on retest after 2 weeks. Although the kappa values were rather low, 40% of subjects classified to gentleness BC type still had the same classification after two weeks, supporting fair reproducibility. The reproducibility of the specific imbalanced BC types was rather low, probably because of the overlapping BC types. The use of a single cut-off score, which may result in the change in the classification with a slight change in the response to scale items. It was also possible that subjects could have a change in their conditions although we only included patients whose reason for visiting the clinic was routine followup of their chronic conditions.

### 5.6. Sensitivity and Responsiveness

The CCMQ (HK versions) showed a difference in the severity of BC types between gender and age groups, supporting their sensitivity. It is likely that it could also differentiate between the healthy, not yet ill, and ill subjects. Further studies on people with different conditions should be carried out to establish its sensitivity in detecting difference in BC types associated with specific illnesses.

This study was the first to investigate and confirm the responsiveness of CCMQ to change in BC type classification and severity. The results also supported the TCM theory that BC type was not static and could change with season and may be subject for health promotion. It was good to note that a higher proportion of subjects changed back to gentleness BC type, and more people changed from “positive” to “negative” for any particular imbalanced BC types on followup. This could be related to an improvement in the health of these clinic patients after their consultations or a seasonal effect. In TCM theory, certain BC types occur more frequently or may become more severe during each season, for example, phlegm-wetness is expected to be more common in the summer than winter, while patients with Yang-deficiency will get more severe during winter but not in summer. Further epidemiological studies on the general population should be carried out to determine the effect of season on BC types. 

### 5.7. Limitations

The subjects were convenient patient samples, and the response rate in the psychometric study was relatively low, which might have biased the distributions of BC types. However, our study samples included people from all age groups and both genders, so the results on validity and reliability of CCMQ should be generalizable to other Chinese people in Hong Kong. The CCMQ were administered by an interviewer to all subjects, so the results might not be applicable to self-administration. No standardization of the CMP diagnosis of BC type was made, which might have affected the accuracy of the predictive sensitivity and specificity of the CCMQ. Better standardization of the CMP diagnosis and assessment of each subject by more than one CMP should be considered in future studies on the accuracy of CCMQ. 

## 6. Conclusions

The CCMQ was adapted to a HK version with some changes to the wording of four items for Cantonese speaking Chinese people in Hong Kong. The construct validity, reliability, sensitivity, and responsiveness of the CCMQ scales were satisfactory. The CCMQ was able to classify the majority of people into one or more BC types. The instrument is useful in differentiating people with the gentleness BC type from those with imbalanced BC types, but the significance of more than one imbalanced BC types needs to be confirmed. One weakness is the relatively low sensitivity and specificity in predicting the CMP BC type diagnosis and low reproducibility in specific BC type classification. The CCMQ has the potential applications in population-based epidemiological studies as well as clinical trials.

Further research should also be done to explore whether the CCMQ can be shortened to improve its acceptability. Calibration of the cut-off scores for the definition of specific BC type should be carried out against better assured gold standards. The performance as an outcome measure in health promotion interventions should be evaluated.

## Supplementary Material

Cognitive debriefing interview table documentation.

## Figures and Tables

**Table 1:** Patients' baseline characteristics.

	Cognitive debriefing patients	Patients		Hong Kong general population (2010)^a^	
	(*n* = 10)	(*n* = 1,084)		(*n* = 7,067,800)	
Age (Year, mean ± SD)	48.±14.9	48.9 ± 14.8		NA	
Age group (*n* (%))*	
18–44 years	5 (50%)	376 (34.7%)		2,875,380 (48.3%)	
45–64 years	3 (30%)	562 (51.9%)		2,162,400 (36.3%)	
>65 years	2 (20%)	140 (12.9%)		912,100 (15.3%)	
Refused to answer	0	5 (0.5%)		NA	
Sex (*n* (%))*	
Male	5 (50%)	324 (29.9%)		3,307,730 (46.8%)	
Female	5 (50%)	756 (69.8%)		3,760,070 (53.2%)	
Marital status (*n* (%))*	
Married living with spouse	NA	683 (63.1%)		3,392,300 (57.6%)	
Single	NA	282 (26.0%)		1,894,100 (32.2%)	
Widower and separated/divorced	NA	115 (10.6%)		601,900 (10.2%)	
Refused to answer	NA	3 (0.3%)		NA	
Education (*n* (%))*	
Nil	NA	45 (4.2%)		321,294 (5.4%)	
Primary	NA	170 (15.7%)		1,005,530 (16.9%)	
Secondary	NA	488 (45.1%)		3,105,837 (52.2%)	
Tertiary	NA	139 (12.8%)		434,341 (7.3%)	
Refused to answer	NA	241 (22.3%)		NA	

		(*n* = 1080)			
		Mean	SD	Floor%	Ceiling%

CCMQ scores					
Gentleness	NA	61.37	16.74	0	0.46
Qi-deficiency	NA	33.62	17.52	1.30	0.09
Yang-deficiency	NA	27.01	21.48	11.79	0.19
Yin-deficiency	NA	26.67	16.64	5.10	0
Phlegm-wetness	NA	26.81	17.42	4.82	0
Wetness-heat	NA	26.36	17.01	7.88	0
Blood-stasis	NA	27.74	16.24	3.25	0
Qi-depression	NA	26.86	18.85	9.94	0
Special diathesis	NA	24.50	17.09	4.91	0
SF-12v2 (HK norm)	
PF (87.6)	NA	83.24	25.24	2.43	61.72
RP (80.0)	NA	75.59	26.83	2.23	38.88
BP (78.1)	NA	69.12	29.31	4.65	35.97
GH (48.3)	NA	34.63	20.95	11.01	0.84
VT (62.6)	NA	55.29	26.45	6.77	9.46
SF (82.0)	NA	81.97	24.38	2.41	55.24
RE (77.4)	NA	77.18	24.46	1.30	38.38
MH (69.1)	NA	68.71	20.37	0.84	11.84
PCS	NA	46.19	9.94	0	0
MCS	NA	50.34	10.58	0	0

PF: physical functioning; RP: role limitation due to physical problems; BP: bodily pain; GH: general health; VT: vitality; SF: social functioning; RE: role limitation due to emotional problems; MH: mental health; PCS: physical component score; MCS: mental component score; NA: not applicable.

*Significant difference between patients and Hong Kong general population by chi-square test.

**Table 2:** The CVIs of CCMQ rated by Chinese medicine practitioners (CMPs) and lay persons.

	CMP (*n* = 10) (%)	Lay persons (*n* = 10) (%)
Scale (Items) rated to be clear		
Response options “often” (*Chang Chang*)	**50**	100
Gentleness (total 8 items)	70–100	50–100
“53. Are you capable to adapt to the external changes of the natural and social environment?”	70	**50**
Qi-deficiency (total 8 items)	60–100	60–100
“3. Will you easily feeling shortness of breath (gasping for breath)?”	**60**	100
“4. Will you easily feeling palpitation?”	90	**60**
Yang-deficiency (total 7 items)	80–100	100
Ying-deficiency (total 8 items)	80–100	90–100
Phlegm-wetness (total 8 items)	80–100	70–100
Wetness-heat (total 6 items)	90–100	60–100
“60. Have you feeling wetness in your scrotum (for men only)?”	100	**60**
Blood stasis (total 7 items)	80–100	90–100
Qi-depression (total 7 items)	60–100	90–100
“10. Do you think you are sentimental or fragile emotionally?”	**60**	100
Special diathesis (total items: 7)	80–100	80–100
Scales rated to be consistent with response options		
Gentleness (total 8 items)	80–100	80–90
Qi-deficiency (total 8 items)	80–100	80–90
Yang-deficiency (total 7 items)	90–100	90
Ying-deficiency (total 8 items)	90–100	90
Phlegm-wetness (total 8 items)	90–100	80–90
Wetness-heat (total 6 items)	100	80–100
Blood stasis (total 7 items)	90–100	70–90
Qi-depression (total 7 items)	90–100	90
Special diathesis (total 7 items)	80–100	80–90
Scales rated to be relevant to health		
Gentleness (total 8 items)	70–100	40–100
“53. Are you capable to adapt to the external changes of the natural and social environment?”	70	**40**
Qi-deficiency (total 8 items)	80–100	50–100
“6. Do you like quiet and bothered to talk?”	80	**50**
Yang-deficiency (total 7 items)	80–100	90–100
Ying-deficiency (total 8 items)	70–100	70–100
Phlegm-wetness (total 8 items)	80–100	70–100
Wetness-heat (total 6 items)	80–100	70–100
Blood stasis (total 7 items)	80–100	70–100
Qi-depression (total 7 items)	80–100	50–100
“14. Will you sign without any intention?”	80	**50**
Special diathesis (total 7 items)	80–100	70–100

**Table 3:** The CCMQ draft version and the final CCMQ (HK) version.

Draft Hong Kong version	Final Hong Kong version
Items	

“3. Will you easily feeling shortness of breath (gasping for breath)?” *(Nin Rong Yi Qi Duan (Hu Xi Duan Cu, Jie Bu Shang Qi) Ma?) *	“3. Will you easily feeling shortness of breath *(gasping for breath)?” (Nin Rong Yi Gan Dao Bu Gou Qi (Hu Xi Ji Cu, Shang Qi Bu Jie Xia Qi) Ma?) *

“26. Will you easily sweat excessively with only a slight movement?” *(Nin Huo Dong Liang Shao Da Jiu Rong Yi Chu Xu Han Ma?) *	“26. Will you sweat excessively with only a slight movement (it seems you sweat more than others)?” *(Ni Huo Dong Liang Shao Da Jiu Rong Yi Chu Xu Han (Bi Yi Ban Ren Rong Yi Chu Hao Duo Han) Ma?) *

“31. Will you easily having skin urticaria (wheal or wind knots)?” *(Nin De Pi Fu Rong Yi Qi Xun Ma Zhen (Feng Tuan, Feng Zhen Kuai, Feng Ge Da) Ma?) *	“31. Will your skin easily having rubella (including wind groups, gives or wind knots)” *(Ni De Pi Fu Rong Yi Qi Feng Zhen (Bao Kuo Feng Tuan, Feng Zhen Kuai, Feng Ge Da) Ma?) *

“32. Will you easily getting skin allergic purpura *(purple blood spots or petechiae)?” (Nin De Pi Fu Yin Guo Min Chu Xian Guo Zi Dian (Zi Hong Se Yu Dian, Yu Ban) Ma?) *	“32. Will you easily getting allergies of purple erythema (purple blood spots or petechiae)?” *(Ni De Pi Fu Win Guo Min Chu Xian Zi Hong Ban (Ji Zi Hong Se Yu Dian, Yu Ban) Ma?) *

Response option	

“Always” *(Jing Chang) *	“For most of the time” *(Da Bu Fen Shi Jian) *

**Table 4:** The distribution of body constitution classified by CCMQ.

	Frequency (*n*/%)		
	Baseline (*n* = 1080)	2 weeks (*n* = 219)	3–6 months (*n* = 390)
Body constitution classification by CCMQ*			
(1) Gentleness	215 (20.0%)	112 (50.5%)	143 (36.7%)
(2) Qi-deficiency	607 (56.2%)	63 (28.1%)	111 (28.2%)
(3) Yang-deficiency	420 (39.0%)	40 (18.1%)	114 (29.2%)
(4) Yin-deficiency	439 (40.7%)	31 (14.0%)	94 (24.1%)
(5) Phlegm-wetness	444 (41.2%)	40 (18.0%)	79 (20.3%)
(6) Wetness-heat	411 (38.1%)	35 (15.9%)	82 (21.0%)
(7) Blood-stasis	432 (40.1%)	44 (19.9%)	103 (26.4%)
(8) Qi-depression	451 (41.9%)	37 (16.7%)	82 (20.9%)
(9) Special diathesis	371 (34.4%)	36 (16.2%)	64 (16.4%)
(10) No body constitution	15 (1.4%)	2 (0.9%)	8 (2.1%)
Number of body constitution by CCMQ			
0	15 (1.4%)	2 (0.9%)	8 (2.0%)
1	364 (33.7%)	132 (58.2%)	217 (53.7%)
2	120 (11.1%)	35 (14.7%)	46 (11.4%)
3	103 (9.5%)	17 (7.6%)	40 (9.9%)
4	98 (9.1%)	10 (4.4%)	21 (5.2%)
5	91 (8.4%)	6 (2.7%)	24 (5.9%)
6	90 (8.3%)	8 (3.6%)	11 (2.7%)
7	102 (9.4%)	5 (2.2%)	11 (2.7%)
8	97 (9.0%)	4 (1.8%)	12 (3.0%)

*Multiple body constitutions in CCMQ were allowed giving percentage >100%.

“No body constitution” means that all body constitutions are “NO.”

**Table 5:** Spearman item-scale correlations and scaling success rate of the CCMQ.

	Baseline (*n* = 1080)	
CCMQ diagnosis	Item-scale correlation	Scaling success rate^†^
(1) Gentleness	0.377–0.686	100%
(2) Qi-deficiency	0.433–0.717	87.5%
(3) Yang-deficiency	0.517–0.811	85.7%
(4) Yin-deficiency	0.475–0.701	100%
(5) Phlegm-wetness	0.531–0.679	87.5%
(6) Wetness-heat	0.563–0.666	100%
(7) Blood-stasis	0.460–0.681	85.7%
(8) Qi-depression	0.488–0.820	85.7%
(9) Special diathesis	0.488–0.712	100%

All item-scale Spearman correlations were significant (*P* < 0.001) for two items.

^†^Scaling success means the item and hypothesized-scale correlation was higher than all items and competing-scale correlations. This rate was the proportion of total number of comparisons for all the items in each scale that was successful.

**Table 6:** Confirmatory factor analysis of CCMQ.

Factor 1			Factor 2			Factor 3		
Gentleness			Qi-deficiency			Yang-deficiency		
Variable	Factor loading	*R* ^2^	Variable	Factor loading	*R* ^2^	Variable	Factor loading	*R* ^2^
*Q1 *	0.497	0.247	*Q2 *	−1.398	0.485	*Q17 *	0.722	0.521
*Q2 *	−2.087	0.485	*Q3 *	0.694	0.481	*Q18 *	0.739	0.546
*Q7 *	−1.602	0.441	*Q4 *	0.713	0.508	*Q19 *	0.755	0.57
*Q8 *	0.804	0.79	*Q5 *	0.678	0.46	*Q21 *	1.253	0.9
*Q21 *	0.474	0.9	*Q6 *	0.449	0.202	*Q22 *	0.466	0.417
*Q27 *	−0.461	0.206	*Q7 *	−0.943	0.441	*Q52 *	0.651	0.424
*Q53 *	0.224	0.05	*Q22 *	0.224	0.217	*Q55 *	0.625	0.391
*Q54 *	−0.485	0.235	*Q26 *	0.362	0.131			

Factor 4			Factor 5			Factor 6		
Yin-deficiency			Phlegm-wetness			Wetness-heat		
Variable	Factor loading	*R* ^2^	Variable	Factor loading	*R* ^2^	Variable	Factor loading	*R* ^2^

*Q16 *	0.602	0.362	*Q13 *	0.697	0.486	*Q39 *	0.539	0.29
*Q20 *	0.658	0.433	*Q15 *	0.713	0.509	*Q41 *	0.568	0.322
*Q29 *	0.507	0.257	*Q28 *	0.523	0.274	*Q48 *	0.582	0.338
*Q35 *	0.665	0.443	*Q42 *	0.588	0.346	*Q56 *	0.612	0.374
*Q38 *	0.651	0.423	*Q49 *	0.689	0.475	*Q59 *	0.591	0.349
*Q44 *	0.629	0.395	*Q50 *	0.456	0.208	*Q60 *	0.554	0.307
*Q46 *	0.651	0.424	*Q51 *	0.475	0.226			
*Q57 *	0.5	0.25	*Q58 *	0.605	0.366			

Factor 7			Factor 8			Factor 9		
Blood-stasis			Qi-depression			Special diathesis		
Variable	Factor loading	*R* ^2^	Variable	Factor loading	*R* ^2^	Variable	Factor loading	*R* ^2^

*Q27 *	−0.009	0.206	*Q*8	1.581	0.79	*Q23 *	0.466	0.217
*Q33 *	0.564	0.318	*Q*9	0.79	0.624	*Q24 *	0.59	0.349
*Q36 *	0.674	0.454	*Q*10	0.806	0.65	*Q25 *	0.689	0.475
*Q37 *	0.443	0.196	*Q*11	0.826	0.682	*Q30 *	0.689	0.475
*Q40 *	0.672	0.451	*Q*12	0.636	0.404	*Q31 *	0.605	0.365
*Q43 *	0.599	0.359	*Q*14	0.676	0.458	*Q32 *	0.621	0.386
*Q45 *	0.651	0.424	*Q*34	0.7	0.49			
			*Q*47	0.612	0.374			

Confirmatory factor analysis model (Goodness of fit statistics for CCMQ)								
SB *χ* ^2^	df	*P* value	RMSEA	90% CI for RMSEA	SRMR	CFI	TLI	IFI

7027.2	1668	<0.001	0.0563	(0.0549, 0.0576)	0.0626	0.969	0.967	0.969

SB *χ* ^2^: The Satorra-Bentler scaled Chi-square statistic; df*:* Degree of freedom; RMSEA: Root mean square error of approximation; SRMR: Standardized root mean square residual; CFI: Comparative fit index; TLI: Tucker-Lewis index; IFI: Incremental Fit index.

**Table 7:** Correlations between the domain scores of the CCMQ and SF-12v2.

(*n* = 1080)	Domains of SF-12									
	PF	RP	BP	GH	VT	SF	RE	MH	PCS	MCS
(1) Gentleness	0.296	0.464	0.442	0.471	0.606	0.442	0.512	0.508	0.368	0.565
(2) Qi-deficiency	−0.317	−0.463	−0.420	−0.394	−0.509	−0.417	−0.483	−0.452	−0.362	−0.499
(3) Yang-deficiency	−0.215	−0.350	−0.338	−0.294	−0.367	−0.326	−0.348	−0.304	−0.294	−0.351
(4) Yin-deficiency	−0.189	−0.354	−0.309	−0.301	−0.343	−0.300	−0.402	−0.383	−0.242	−0.410
(5) Phlegm-wetness	−0.240	−0.395	−0.374	−0.346	−0.377	−0.349	−0.397	−0.393	−0.309	−0.403
(6) Wetness-heat	−0.112	−0.283	−0.272	−0.208	−0.281	−0.287	−0.318	−0.297	−0.179	−0.342
(7) Blood-stasis	−0.260	−0.392	−0.395	−0.368	−0.399	−0.337	−0.397	−0.382	−0.342	−0.397
(8) Qi-depression	−0.228	−0.394	−0.359	−0.380	−0.451	−0.426	−0.532	−0.601	−0.233	−0.612
(9) Special diathesis	−0.152	−0.275	−0.248	−0.187	−0.249	−0.261	−0.273	−0.282	−0.193	−0.292

PF: physical functioning; RP: role limitation due to physical problems; BP: bodily pain; GH: general health; VT: vitality; SF: social functioning; RE: role limitation due to emotional problems; MH: mental health; PCS: physical component score; MCS: mental component score.

All spearman correlations are significant, *P* < 0.01.

**Table 8:** SF-12v2 scores by body constitutions types of CCMQ.

Baseline (*n* = 1080)	SF12V2 mean (sd)									
	PF	RP	BP	GH	VT	SF	RE	MH	PCS	MCS
(1) Gentleness	90.8 (20.7)	88.1 (21.2)	84.0 (24.8)	45.9 (21.4)	71.3 (23.4)	93.3 (15.5)	91.2 (16.4)	81.8 (16.2)	49.8 (8.2)	57.2 (8.2)
(2) Qi-deficiency	78.1 (27.4)	67.5 (27.8)	60.8 (28.6)	29.2 (19.4)	46.2 (24.9)	75.2 (26.5)	69.1 (25.8)	62.1 (20.1)	43.9 (10.5)	46.6 (10.6)
(3) Yang-deficiency	78.0 (28.0)	65.7 (29.0)	59.4 (29.1)	28.6 (19.6)	45.8 (26.0)	73.5 (26.8)	68.8 (26.0)	62.9 (21.1)	43.3 (10.5)	46.7 (10.7)
(4) Yin-deficiency	78.6 (27.1)	66.2 (27.8)	60.2 (28.9)	27.9 (19.4)	46.7 (25.9)	75.1 (26.5)	67.2 (25.8)	61.4 (19.8)	43.8 (10.4)	46.2 (10.6)
(5) Phlegm-wetness	76.5 (28.4)	65.4 (27.8)	58.9 (28.2)	27.9 (18.8)	45.4 (25.1)	74.0 (26.9)	67.7 (26.4)	61.8 (19.9)	43.0 (10.6)	46.5 (10.9)
(6) Wetness-heat	81.0 (26.6)	68.5 (27.9)	62.3 (28.3)	30.3 (19.5)	47.4 (25.3)	75.3 (26.6)	70.5 (25.9)	63.9 (19.0)	44.5 (10.2)	47.1 (10.6)
(7) Blood-stasis	77.9 (27.1)	66.2 (27.9)	59.6 (27.5)	27.5 (18.8)	45.5 (25.2)	74.5 (26.5)	67.8 (26.5)	61.2 (20.4)	43.5 (10.1)	46.2 (10.7)
(8) Qi-depression	78.1 (27.4)	67.7 (27.8)	63.1 (28.7)	29.6 (19.9)	48.5 (25.2)	75.1 (26.8)	69.4 (26.8)	62.7 (20.2)	44.1 (10.5)	46.9 (10.8)
(9) Special diathesis	77.7 (27.2)	64.9 (28.3)	58.9 (27.9)	26.7 (17.4)	43.9 (25.3)	71.9 (27.3)	64.6 (25.4)	57.4 (19.2)	43.8 (10.2)	44.3 (10.3)

PF: physical functioning; RP: role limitation due to physical problems; BP: bodily pain; GH: general health; VT: vitality; SF: social functioning; RE: role limitation due to emotional problems; MH: mental health; PCS: physical component score; MCS: mental component score.

Difference between mean SF-12v2 scores of gentleness and imbalanced physiological constitution groups are statistically significant by independent *t*-test samples (*P* < 0.05).

**Table 9:** Cronbach's alpha and ICC of the CCMQ.

	Cronbach's alpha (*n* = 1080)	ICC (95% CI) (*n* = 219)
CCMQ	0.89	
(1) Gentleness	0.72	0.78 (0.74, 0.82)
(2) Qi-deficiency	0.76	0.82 (0.78, 0.85)
(3) Yang-deficiency	0.82	0.88 (0.86, 0.90)
(4) Yin-deficiency	0.75	0.80 (0.75, 0.83)
(5) Phlegm-wetness	0.74	0.78 (0.74, 0.82)
(6) Wetness-heat	0.66	0.76 (0.71, 0.80)
(7) Blood-stasis	0.67	0.71 (0.65, 0.76)
(8) Qi-depression	0.84	0.85 (0.82, 0.88)
(9) Special diathesis	0.74	0.81 (0.77, 0.85)

**Table 10:** Reliability of diagnosis after 2-week followup of CCMQ.

	At baseline	After 2-week followup		Kappa statistics
		No	Yes	
CCMQ diagnosis				

(1) Gentleness	No (*n* = 160)	96 (60.0%)	64 (40.0%)	0.318
	Yes (*n* = 58)	11 (19.0%)	47 (81.0%)	
(2) Qi-deficiency	No (*n* = 116)	112 (96.6%)	4 (3.4%)	0.531
	Yes (*n* = 106)	47 (44.3%)	59 (55.7%)	
(3) Yang-deficiency	No (*n* = 148)	142 (95.9%)	6 (4.1%)	0.488
	Yes (*n* = 70)	37 (52.9%)	33 (47.1%)	
(4) Yin-deficiency	No (*n* = 148)	141 (95.3%)	7 (4.7%)	0.346
	Yes (*n* = 70)	46 (65.7%)	24 (34.3%)	
(5) Phlegm-wetness	No (*n* = 141)	133 (94.3%)	8 (5.7%)	0.397
	Yes (*n* = 78)	46 (59.0%)	32 (41.0%)	
(6) Wetness-heat	No (*n* = 149)	139 (93.3%)	10 (6.7%)	0.340
	Yes (*n* = 69)	44 (63.8%)	25 (36.2%)	
(7) Blood-stasis	No (*n* = 146)	135 (92.5%)	11 (7.5%)	0.411
	Yes (*n* = 72)	40 (55.6%)	32 (44.4%)	
(8) Qi-depression	No (*n* = 140)	133 (95.0%)	7 (5.0%)	0.373
	Yes (*n* = 79)	49 (62.0%)	30 (38.0%)	
(9) Special diathesis	No (*n* = 151)	142 (94.0%)	9 (6.0%)	0.372
	Yes (*n* = 68)	42 (61.8%)	26 (38.2%)	

**Table 11:** Chinese medicine practitioners diagnosis against CCMQ classification of body constitutions.

		CMP		Sensitivity	Specificity
		Yes	No		
CCMQ					

(1) Gentleness	Yes (*n* = 204)	34	170	54.8	82.7
	No (*n* = 841)	28	813		
(2) Qi-deficiency	Yes (*n* = 593)	69	524	51.9	42.7
	No (*n* = 455)	64	391		
(3) Yang-deficiency	Yes (*n* = 412)	65	347	48.5	61.9
	No (*n* = 633)	69	564		
(4) Yin-deficiency	Yes (*n* = 429)	101	328	44.5	60.0
	No (*n* = 617)	126	491		
(5) Phlegm-wetness	Yes (*n* = 432)	82	350	43.4	59.2
	No (*n* = 614)	107	507		
(6) Wetness-heat	Yes (*n* = 407)	45	362	42.9	61.6
	No (*n* = 641)	60	581		
(7) Blood-stasis	Yes (*n* = 421)	49	372	44.1	60.2
	No (*n* = 625)	62	563		
(8) Qi-depression	Yes (*n* = 440)	40	400	61.5	59.2
	No (*n* = 605)	25	580		
(9) Special diathesis	Yes (*n* = 360)	15	345	75.0	66.4
	No (*n* = 686)	5	681		

**Table 12:** CCMQ and SF-12v2 scores by gender and age groups.

	Gender				Age group					
	Male (*n* = 324)		Female (*n* = 756)		18–40 (*n* = 376)		41–64 (*n* = 562)		>65 (*n* = 140)	
	Mean	SD	Mean	SD	Mean	SD	Mean	SD	Mean	SD
CCMQ										

(1) Gentleness^∗†^	66.9	15.6	59.0	16.7	58.5	16.4	61.6	16.8	68.8	14.9
(2) Qi-deficiency^∗†^	29.3	15.9	35.5	17.8	37.7	17.1	33.6	17.2	22.5	14.9
(3) Yang-deficiency^∗†^	18.1	16.7	30.9	22.2	31.5	21.3	26.1	21.4	17.7	18.5
(4) Yin-deficiency^∗†^	21.8	15.9	28.7	16.5	31.5	16.3	26.2	16.4	15.6	12.7
(5) Phlegm-wetness^∗†^	23.2	16.4	28.3	17.6	29.6	17.2	27.5	17.5	16.7	14.2
(6) Wetness-heat^†^	26.4	16.3	26.3	17.3	33.4	17.1	24.3	15.7	15.5	13.9
(7) Blood-stasis^∗†^	20.0	13.6	31.1	16.1	31.0	16.3	28.0	16.3	18.4	11.5
(8) Qi-depression^∗†^	20.6	17.1	29.6	18.9	31.6	18.2	26.5	18.9	15.2	14.9
(9) Special diathesis^∗†^	21.8	16.0	25.6	17.4	29.1	17.6	23.7	16.4	15.6	14.3
SF-12v2										
(1) Physical component score^∗†^	48.6	8.5	45.2	10.3	47.6	9.0	45.8	9.9	44.1	11.8
(2) Mental component score	51.4	9.9	49.9	10.8	47.6	10.0	50.9	10.4	55.7	10.1

*Significant difference between the patients with gender by Mann-Whitney *U* test (*P* < 0.05).

^†^Significant difference between the patients with age by Kruskal-Wallis *H* test (*P* < 0.05).

**Table 13:** Change of CCMQ at baseline and after 3–6-month followup.

	Baseline		After 3–6-month followup		Body constitution at baseline	Body constitution after 3–6-month followup	
	Mean	SD	Mean	SD		Yes	No
(1) Gentleness^∗†^	60.1	16.7	67.4	18.3	Yes (*n* = 68)	54 (79.4%)	14 (20.6%)
					No (*n* = 330)	91 (27.6%)	239 (72.4%)
(2) Qi-deficiency^∗†^	35.0	17.9	23.9	18.2	Yes (*n* = 232)	109 (47.0%)	123 (53.0%)
					No (*n* = 171)	8 (4.7%)	163 (95.3%)
(3) Yang-deficiency^∗†^	27.6	22.1	22.1	21.2	Yes (*n* = 156)	94 (60.3%)	62 (39.7%)
					No (*n* = 242)	25 (10.3%)	217 (89.7%)
(4) Yin-deficiency^∗†^	27.1	16.6	20.6	14.8	Yes (*n* = 159)	80 (50.3%)	79 (49.7%)
					No (*n* = 240)	18 (7.5%)	222 (92.5%)
(5) Phlegm-wetness^∗†^	28.2	17.4	18.9	15.2	Yes (*n* = 180)	72 (40.0%)	108 (60.0%)
					No (*n* = 219)	10 (4.6%)	209 (95.4%)
(6) Wetness-heat^∗†^	26.7	17.3	19.7	15.1	Yes (*n* = 154)	70 (45.5%)	84 (54.5%)
					No (*n* = 245)	16 (6.5%)	229 (93.5%)
(7) Blood-stasis^∗†^	28.5	15.9	22.5	15.2	Yes (*n* = 168)	86 (51.2%)	82 (48.8%)
					No (*n* = 231)	20 (8.7%)	211 (91.3%)
(8) Qi-depression^∗†^	28.3	19.4	19.1	17.7	Yes (*n* = 178)	75 (42.1%)	103 (57.9%)
					No (*n* = 223)	12 (5.4%)	211 (94.6%)
(9) Special diathesis^∗†^	25.4	17.2	17.8	13.1	Yes (*n* = 145)	55 (37.9%)	90 (62.1%)
					No (*n* = 254)	13 (5.1%)	241 (94.9%)

SF-12v2							
(1) Physical component score^†^	46.0	10.7	48.6	9.6			
(2) Mental component score^†^	49.9	10.9	52.5	10.6			

*Significant difference between baseline and 3–6-month followup by paired *t*-test.

^†^Significant difference between baseline and 3–6-month followup by McNemar-Bowker test.

## References

[1] Birkeflet O, Laake P, Vøllestad N (2011). Low inter-rater reliability in traditional Chinese medicine for female infertility. *Acupuncture in Medicine*.

[2] Mist S, Ritenbaugh C, Aickin M (2009). Effects of questionnaire-based diagnosis and training on inter-rater reliability among practitioners of traditional chinese medicine. *Journal of Alternative and Complementary Medicine*.

[3] O’Brien KA, Abbas E, Zhang J (2009). An investigation into the reliability of Chinese medicine diagnosis according to eight guiding principles and Zang-Fu theory in Australians with hypercholesterolemia. *Journal of Alternative and Complementary Medicine*.

[4] Hao YT, Fanc JQ (2000). The introduce and usage of WHOQOL instrument in chinese. *Modern Rehabilitation*.

[5] Liu F-B, Fang J-Q, Pan Z-H (2000). The development of syndrome differential scale of the spleen-stomach diseases used for computer aided expert diagnosis system. *Academic Journal of Sun Yat-Sen University of Medical Sciences*.

[6] Wang Q (2005). Classification and diagnosis basis of nine basic constitutions in Chinese medicine. *Journal of Beijing University of Traditional Chinese Medicine*.

[7] Wang Q (2006). Compiling and application of constitution in Chinese medicine questionnaire. *China Journal of Traditional Chinese Medicine and Pharmacy*.

[8] Ren X-J, Wang Q (2007). Discussion on application of traditional chinese medicine classification standards of health in personal health management. *Chinese Health Service Management*.

[9] Wang Q, Zhu YB, Xue HS, Li S (2006). Primary compiling of constitution in Chinese medicine questionnaire. *Chinese Journal of Clinical Rehabilitation*.

[10] Su Y-C (2007). Establishment of traditional Chinese medical constitutional scale and classificatory index (2-1). *Yearbook of Chinese Medicine and Pharmacy*.

[11] Su Y-C (2009). The evaluation of traditional Chinese medical constitutional scale and classification index. *Yearbook of Chinese Medicine and Pharmacy*.

[12] Wu C (2009). Epidemiological study of 2043 body constitutions based on Chinese medicine theory in jiangsu provision. *Chinese Journal of Basic Medicine in Traditional Chinese Medicine*.

[13] Su YC (2008). The creation of traditional Chinese medical constitutional scale and classification index (2-2). *Yearbook of Chinese Medicine and Pharmacy*.

[14] Lam CLK (2010). Validation of the Chinese (HK) version of the two constitution measurement instruments (BCQ and CCMQ) in Hong Kong. *Third Quarter Chinese Medicine Research Monitoring Report*.

[15] Su Y-C, Chen L-L, Lin J-D, Lin J-S, Huang Y-C, Lai J-S (2008). BCQ+: a body constitution questionnaire to assess Yang-Xu: part I: establishment of a first final version through a Delphi process. *Forschende Komplementarmedizin*.

[16] Chen L-L, Lin J-S, Lin J-D (2009). BCQ+: a body constitution questionnaire to assess Yang-Xu: part II: evaluation of reliability and validity. *Forschende Komplementarmedizin*.

[17] Lin J-D, Chen L-L, Lin J-S, Chang C-H, Huang Y-C, Su Y-C (2012). BCQ: a body constitution questionnaire to assess Yin-Xu. Part I: establishment of a provisional version through a Delphi process. *Forschende Komplementärmedizin*.

[18] Lin J-S, Chen L-L, Lin J-D (2012). BCQ+: a body constitution questionnaire to assess Yang-Xu. Part II: evaluation of reliability and validity. *Forschende Komplementärmedizin*.

[19] Lin J-D, Lin J-S, Chen L-L, Chang C-H, Huang Y-C, Su Y-C (2012). BCQs: a body constitution questionnaire to assess stasis in traditional Chinese medicine. *European Journal of Integrative Medicine*.

[20] Zhu Y-B, Wang Q, Hideki O (2007). Evaluation on reliability and validity of the constitution in Chinese medicine questionnaire (CCMQ). *Chinese Journal of Behavioral Medical Science*.

[21] State Administration of Traditional Chinese Medicine of the People's Republic of China (2008). Introduction of Not-yet-ill health promotion project. *Chinese Journal of Management in Chinese Medicine*.

[22] Wang Q, Zhu Y-B (2009). Epidemiological investigation of constitutional types of Chinese medicine in general population: base on 21, 948 epidemiological investigation data of nine provinces in China. *China Journal of Traditional Chinese Medicine and Pharmacy*.

[23] Wong W, Lam CLK, Leung KF, Zhao L (2009). Is the content of the Chinese quality of life instrument (ChQOL) really valid in the context of traditional Chinese medicine in Hong Kong?. *Complementary Therapies in Medicine*.

[24] Wild D, Grove A, Martin M (2005). Principles of good practice for the translation and cultural adaptation process for patient-reported outcomes (PRO) measures: report of the ISPOR Task Force for Translation and Cultural Adaptation. *Value in Health*.

[25] Rosser RM (1976). Recent studies using a global approach to measuring illness. *Medical Care*.

[26] Nunnally JC, Bernstein IH (1994). *Psychometric Theory*.

[27] Zhu YB, Wang Q, Xue HS, Orikasa Q (2006). Preliminary assessment on performance of constitution in Chinese medicine questionnaire. *Chinese Journal of Clinical Rehabilitation*.

[28] Lam CLK, Tse EYY, Gandek B (2005). Is the standard SF-12 Health Survey valid and equivalent for a Chinese population?. *Quality of Life Research*.

[29] Lynn MR (1986). Determination and quantification of content validity. *Nursing Research*.

[30] Ware JE, Gandek B (1998). Methods for testing data quality, scaling assumptions, and reliability: The IQOLA Project approach. *Journal of Clinical Epidemiology*.

[31] McHorney CA, Tarlov AR (1995). Individual-patient monitoring in clinical practice: are available health status surveys adequate?. *Quality of Life Research*.

[32] Satorra A, Bentler E, von Eye A, Clogg C (1994). Corrections to test statistics and standard enors in covariance structure analysis. *Latent Variables Analysis: Applications For Developmental Research*.

[33] Hu LT, Bentler PM (1999). Cutoff criteria for fit indexes in covariance structure analysis: conventional criteria versus new alternatives. *Structural Equation Modeling*.

[34] Landis JR, Koch GG (1977). The measurement of observer agreement for categorical data. *Biometrics*.

[35] Terwee CB, Bot SDM, de Boer MR (2007). Quality criteria were proposed for measurement properties of health status questionnaires. *Journal of Clinical Epidemiology*.

[36] Shrout PE, Fleiss JL (1979). Intraclass correlations: uses in assessing rater reliability. *Psychological Bulletin*.

[37] Sung JJY, Leung WK, Ching JYL (2004). Agreements among traditional Chinese medicine practitioners in the diagnosis and treatment of irritable bowel syndrome. *Alimentary Pharmacology and Therapeutics*.

