# Supplementary material for: Validation of the Constitution in Chinese Medicine Questionnaire: Does the Traditional Chinese Medicine Concept of Body Constitution Exist?
Source: Evid Based Complement Alternat Med. 2013 Apr 24;2013:481491. doi: 10.1155/2013/481491 (PMC3655622; doi:10.1155/2013/481491)
Supplement: Supplementary file 1 — Cognitive debriefing interview table documentation. [file 481491.f1.pdf]

Appendix A 參加者編號: ____________

HK Version of CCMQ_Cognitive debriefing
中醫體質量表(香港版) 內容理解分析表

問卷內容	你對問卷內容的意見				
	你明白這段文字的意思嗎?	若你不明白這段文字的意思，你認為有那些地方需要修改？	你可否解釋這段文字是什麼意思?	你認為這題的答案與問題配合嗎?	你認為這條問題跟健康有關嗎?
中醫體質量表					
本問卷是為了調查與你的體質有關的一些情況，從而為今後你的健康管理和臨床診治等提供參考。					
請閱讀每條問題，根據自己**<u>最近一年</u>**的實際情況或感覺，圈出最符合你的答案。					
如果你未能肯定如何回答某條問題時，請選擇最接近你實際情況的答案。					
請注意：所有問題都是根據你最近一年的情況作答，而且每條問題只能選擇一個答案。					
請根據最近一年的體驗和感覺，回答以下問題。					
(1) 你精力充沛嗎？					
沒有(或不會)					
很少					
有時					
經常					
常常					
(2) 你容易疲倦嗎？					
(3) 你容易感到不够氣 (呼吸急促， 上氣不接下氣) 嗎？					
(4) 你容易心慌嗎？					
(5) 你容易頭暈或站起時暈眩嗎？					
(6) 你喜歡安靜、懶得說話嗎?					
(7) 你說話的聲音虛弱無力嗎?					
(8) 你感到悶悶不樂、情緒低					

問卷內容	你對問卷內容的意見				
	你明白這段文字的意思嗎?	若你不明白這段文字的意思，你認為有那些地方需要修改？	你可否解釋這段文字是什麼意思?	你認為這題的答案與問題配合嗎?	你認為這條問題跟健康有關嗎?
落嗎?					
(9) 你容易精神緊張、焦慮不安嗎?					
(10) 你多愁善感、感情脆弱嗎？					
(11) 你容易感到害怕或受到驚嚇嗎?					
(12) 你肋骨部位或乳房脹痛嗎?					
(13) 你感到胸悶或腹部脹滿嗎？					
(14) 你會無緣無故歎氣嗎?					
(15) 你感到身體沉重不輕鬆或不爽快嗎?					
(16) 你感到手心或腳掌心發熱嗎?					
(17) 你手腳凍嗎?					
(18) 你胃部、背部或腰膝部位怕冷嗎？					
(19) 你怕冷、衣服**較其他人**穿得多嗎?					
(20) 你感覺身體、臉上發熱嗎？					
(21) 你**比一般人**較不能抵受寒冷 (如冬天的寒冷，夏天的空調冷氣、電扇等) 嗎？					
(22) 你**比其他人較**容易感冒嗎?					
(23) 你**沒有感冒時**也會打噴嚏嗎？					
(24) 你**沒有感冒時**也會鼻					

問卷內容	你對問卷內容的意見				
	你明白這段文字的意思嗎?	若你不明白這段文字的意思，你認為有那些地方需要修改？	你可否解釋這段文字是什麼意思?	你認為這題的答案與問題配合嗎?	你認為這條問題跟健康有關嗎?
塞、流鼻涕嗎?					
(25) 你有**因季節變化、溫度變化或異味等原因**而咳嗽或喘氣的現象嗎？					
(26) 你**活動量稍大**就容易出**虛汗** (比一般人容易出**好多**汗) 嗎?					
(27) 你容易忘記事情（健忘）嗎？					
(28) 你有額頭油脂分泌多的現象嗎?					
(29) 你口唇的顏色**比一般人較**紅嗎?					
(30) 你容易過敏 (對藥物、食物、氣味、花粉或在季節交替、氣候變化時) 嗎?					
(31) 你的皮膚容易起風疹 (包括風團、風疹塊、風疙瘩) 嗎?					
(32) 你的皮膚**因過敏**出現紫紅斑 (即紫紅色瘀點、瘀斑) 嗎?					
(33) 你的皮膚**在不知不覺中**會出現青紫瘀斑 (皮下出血) 嗎?					
(34) 你的皮膚一抓就紅，並出現抓痕嗎?					
(35) 你皮膚或口唇乾嗎?					
(36) 你面上顴骨兩面有細微紅絲嗎?					
(37) 你身體上有哪裡疼痛嗎？					
(38) 你面部**兩顴**泛紅或偏紅嗎?					
(39) 你面部或鼻部有油膩感或者油亮發光嗎?					
(40) 你面色暗淡、或容易出現灰褐色斑嗎?					

問卷內容	你對問卷內容的意見				
	你明白這段文字的意思嗎?	若你不明白這段文字的意思，你認為有那些地方需要修改？	你可否解釋這段文字是什麼意思?	你認為這題的答案與問題配合嗎?	你認為這條問題跟健康有關嗎?
(41) 你易生粉刺、暗瘡或膿瘡嗎?					
(42) 你上眼瞼**比別人較**腫（上眼瞼有輕微隆起的現象）嗎?					
(43) 你容易有黑眼圈嗎?					
(44) 你感到眼睛乾澀嗎？					
(45) 你口唇顏色偏暗嗎?					
(46) 你感到口乾喉燥、總想喝水嗎？					
(47) 你覺得喉嚨部位有異物感，而且吐不出來，也無法吞下嗎？					
(48) 你感到口苦或口腔有異味嗎?					
(49) 你口腔有黏黏的感覺嗎?					
(50) 你腹部肥滿鬆軟嗎?					
(51) 你平時痰多，特別是喉嚨部位總感到有痰堵塞著嗎?					
(52) 你**吃 (喝) 涼的食物**時會感到不舒服或者怕吃 (喝) 涼的食物嗎？					
(53) 你能適應自然和社會環境的外在變化嗎？					
(54) 你容易失眠嗎？					
(55) 你**著涼或吃 (喝) 涼的食物後**，容易腹瀉嗎?					
(56) 你的大便黏糊不爽、有未排清的感覺嗎?					
(57) 你容易便秘或大便乾燥嗎?					
(58) 你舌苔厚膩或有舌苔厚厚的感覺嗎?					
(59) 你小便時尿道發熱、尿色深黃嗎？					
(60) 你的「白帶」顏色偏黃嗎?（**限女性回答**）					

問卷內容	你對問卷內容的意見				
	你明白這段文字的意思嗎?	若你不明白這段文字的意思，你認為有那些地方需要修改？	你可否解釋這段文字是什麼意思?	你認為這題的答案與問題配合嗎?	你認為這條問題跟健康有關嗎?
(60) 你的陰囊部位潮濕嗎?<br>（限男性回答）					

Appendix B 參加者編號：________

Patient initial assessment form

醫院管理局中醫藥部
香港大學家庭醫學部

《測試中文 (香港版) 的中醫體質量表》

病人初診評估量表

參加者姓名：________________

參加日期：________________

覆診日期：________________

電話號碼：________________

訪問員姓名：________________

訪問員編號：________________

填寫方法： 面訪☐ (1) /自填 ☐ (2)

兩週/六個月後願意跟進: 願意 ☐ (1)/不願意 ☐ (2)

參加者編號：________

病人初診評估量表

1. 請問你今天主要為什麼來這診所求診? (例如: 關節痛、咳嗽、月經不調或皮膚病等)
   _______________ (ICPC-2)

2. 你這個病有多久了? _____________(天/月/年)?

3. 請問你以前有沒有為這問題向其他中/西醫求診?
   1. 沒有
   2. 有，向西醫求診
   3. 有，向中醫求診
   4. 有，同時向中和西醫求診

4. 請問你有沒有經由西醫診斷患有任何一種慢性疾病 （多過四星期以上）：
   1. 沒有
   2. 有 (如: 心臟病、血壓高、糖尿病、慢性腎炎心理或精神病等）
      請註明：____________________________
   3. 唔知

5. 請問你有沒有需要長期服用任何藥物？
   1. 沒有
   2. 有，請註明種類 _______________
   3. 唔知

6. 你有固定的工作/上學/作息時間?
   1. 有
      若有的話，上午/下午__________點 ~ 上午/下午__________點
   2. 無

7. 你每天大約什麼時間上床睡覺，什麼時間起床?
   上床時間：_________ 起床時間：__________

8. 你是否經常捱夜(超過 12:00)以後才睡
   1. 無
   2. 偶爾
   3. 經常

9. 你平常喜歡吃那種口味的食物
    1. 無特殊喜好
    2. 酸味
    3. 苦味
    4. 甜味
    5. 辛辣
    6. 鹹味

10. 你是否喜歡吃冰品(冰製品及冰飲料)?
    1. 無
    2. 偶爾
    3. 經常

11. 你是否吃油炸食品?
    1. 無
    2. 偶爾
    3. 經常

12. 性別？
    1. 男
    2. 女

13. 請問你的年齡是：________ 歲 (999 if not willing to tell)

14. 請問你的教育程度是多少？
    1. 從沒有進學
    2. 小學
    3. 中學（包括預科）
    4. 專上學院
    5. 大學或以上
    6. 唔知

15. 請問你的婚姻狀況是？
    1. 已婚，與配偶同住
    2. 已婚，但配偶已去世
    3. 單身
    4. 已分居或離婚
    5. 唔知

16. 請問你的職業是（如果是失業／退休，請問過去職業）
    1. 專業人員

2. 副專業人員
3. 技術工人
4. 半技術工人
5. 非技術工人
6. 主婦，請問一家之主的職業是：____________
7. 學生，請問一家之主的職業是：____________
8. 其他，請註明：______________
9. 拒絕回答

17. 請問你的家庭總收入大約是多少？
    1. <HK$ 4,000
    2. HK$ 4,000 – HK$ 9,999
    3. HK$ 10,000 – HK$ 16,999
    4. HK$ 17,000 – HK$ 26,999
    5. HK$ 27,000 – HK$ 59,999
    6. □HK$ 60,000
    7. 拒絕回答

（初診）

CCMQ 中醫體質量表（香港版）

本問卷是為了調查與你的體質有關的一些情況，從而為今後你的健康管理和臨床診治等提供參考。請閱讀每條問題，根據自己最近一年的實際情況或感覺，圈出最符合你的答案。如果你未能肯定如何回答某條問題時，請選擇最接近你實際情況的答案。

請注意：所有問題都是根據你最近一年的情況作答，而且每條問題只能選擇一個答案。

請根據最近一年的身體狀況和感覺，回答以下問題。	沒有(或不會)	很少	有時	大部份時間	常常
(1) 你精力充沛嗎？	1	2	3	4	5
(2) 你容易疲倦嗎？	1	2	3	4	5
(3) 你容易感到不够氣 (呼吸急促， 上氣不接下氣) 嗎？	1	2	3	4	5
(4) 你容易心慌嗎 “(突然感覺到心跳明顯/心跳加速)” ？	1	2	3	4	5
(5) 你容易頭暈或站起時暈眩嗎？	1	2	3	4	5
(6) 你喜歡安靜、懶得說話嗎?	1	2	3	4	5
(7) 你說話的聲音虛弱無力嗎?	1	2	3	4	5
(8) 你感到悶悶不樂、情緒低落嗎?	1	2	3	4	5

Appendix B 參加者編號：________

請根據最近一年的體驗和感覺，回答以下問題。	**沒有 (或不會)**	**很少**	**有時**	**經常**	**常常**
(9) 你容易精神緊張、焦慮不安嗎?	1	2	3	4	5
(10) 你多愁善感、容易感到悲傷嗎？	1	2	3	4	5
(11) 你容易感到害怕或受驚嗎?	1	2	3	4	5
(12) 你肋骨部位或乳房脹痛嗎?	1	2	3	4	5
(13) 你感到胸口翳悶或腹部脹滿嗎？	1	2	3	4	5
(14) 你會無緣無故歎氣嗎?	1	2	3	4	5
(15) 你感到身體沉重不輕鬆嗎?	1	2	3	4	5
(16) 你感到手心或腳掌心發熱嗎?	1	2	3	4	5
(17) 你手腳凍嗎?	1	2	3	4	5
(18) 你胃部、背部或腰膝部位怕冷嗎？	1	2	3	4	5
(19) 你怕冷、衣服**較其他人**穿得多嗎?	1	2	3	4	5
(20) **你沒有生病的情況下,** 你會感到身體、臉上發熱嗎？	1	2	3	4	5
(21) 你**比一般人**較不能抵受寒冷(如冬天的寒冷，夏天的空調冷氣、電扇等)嗎？	1	2	3	4	5
(22) 你**比其他人**較容易感冒嗎?	1	2	3	4	5

Appendix B 參加者編號：________

<u>請根據最近一年的體驗和感覺，回答以下問題。</u>	**沒有 (或不會)**	**很少**	**有時**	**經常**	**常常**
(23) 你**<u>沒有感冒時</u>**也會打噴嚏嗎？	1	2	3	4	5
(24) 你**<u>沒有感冒時</u>**也會鼻塞、流鼻涕嗎?	1	2	3	4	5
(25) 你有**<u>因季節變化、溫度變化或異味等原因</u>**而咳嗽或喘氣的現象嗎？	1	2	3	4	5
(26) 你**<u>活動量稍大</u>**就容易出**<u>虛汗</u>**嗎?<br>(虛汗的意思是指比一般人容易大量出汗)	1	2	3	4	5
(27) 你容易忘記事情（健忘）嗎？	1	2	3	4	5
(28) 你有額頭油脂分泌多的現象嗎?	1	2	3	4	5
(29) 你口唇的顏色**<u>比一般人</u>**較紅嗎?	1	2	3	4	5
(30) 你容易過敏(對藥物、食物、氣味、花粉或在季節交替、氣候變化時)嗎?	1	2	3	4	5
(31) 你的皮膚容易起風疹(包括風團、風疹塊、風疙瘩)嗎?	1	2	3	4	5
(32) 你的皮膚**<u>因過敏</u>**出現紫红斑(即紫紅色瘀點、瘀斑)嗎?	1	2	3	4	5
(33) 你的皮膚**<u>在不知不覺中</u>**會出現青紫瘀斑(皮下出血)嗎?	1	2	3	4	5
(34) 你的皮膚一抓就紅，並出現抓痕嗎?	1	2	3	4	5
(35) 你皮膚或口唇乾嗎?	1	2	3	4	5
(36) 你面上顴骨兩面有細微紅絲嗎?	1	2	3	4	5

Appendix B 參加者編號：________

請根據最近一年的體驗和感覺，回答以下問題。	沒有(或不會)	很少	有時	經常	常常
(37) 你身體上有哪裡疼痛嗎？	1	2	3	4	5
(38) 你面部**兩顴**泛紅或偏紅嗎?	1	2	3	4	5
(39) 你面部或鼻部有油膩感或者油亮發光嗎?	1	2	3	4	5
(40) 你面色暗淡、或容易出現灰褐色斑嗎?	1	2	3	4	5
(41) 你易生粉刺、暗瘡或膿瘡嗎?	1	2	3	4	5
(42) 你上眼瞼**比別人**較腫（上眼瞼有輕微隆起的現象）嗎?	1	2	3	4	5
(43) 你容易有黑眼圈嗎?	1	2	3	4	5
(44) 你感到眼睛乾澀嗎？	1	2	3	4	5
(45) 你口唇顏色偏暗嗎?	1	2	3	4	5
(46) 你感到口乾喉燥、總想喝水嗎？	1	2	3	4	5
(47) 你覺得喉嚨部位有異物感，而且吐不出來，也無法吞下嗎？	1	2	3	4	5
(48) 你感到口苦或口腔有異味嗎?	1	2	3	4	5
(49) 你口腔有黏黏的感覺嗎?	1	2	3	4	5
(50) 你腹部肥滿鬆軟嗎?	1	2	3	4	5

Appendix B 參加者編號：________

請根據最近一年的體驗和感覺，回答以下問題。	**沒有 (或不會)**	**很少**	**有時**	**經常**	**常常**
(51) 你平時痰多，特別是喉嚨部位總感到有痰堵塞著嗎?	1	2	3	4	5
(52) 你**吃(喝)涼的食物**時會感到不舒服或者怕吃(喝)涼的食物嗎？	1	2	3	4	5
(53) 你能適應自然和社會環境的外在變化嗎？	1	2	3	4	5
(54) 你容易失眠嗎？	1	2	3	4	5
(55) 你**著涼或吃(喝)涼的食物後**，容易腹瀉嗎?	1	2	3	4	5
(56) 你的大便黏糊不爽、有未排清的感覺嗎?	1	2	3	4	5
(57) 你容易便秘或大便乾燥嗎?	1	2	3	4	5
(58) 你舌苔厚膩或有舌苔厚厚的感覺嗎?	1	2	3	4	5
(59) 你小便時尿道發熱、尿色深黃嗎？	1	2	3	4	5
(60) 你的「白帶」顏色偏黃嗎?（**限女性回答**）	1	2	3	4	5
(60) 你的陰囊部位潮濕嗎?（**限男性回答**）	1	2	3	4	5

 參加者編號：________

A-7. 跟您三個月前相比，您認為您的健康情況有多少改變？

好了很多	好了一半	好了一點	沒有轉變	差了一點	差了一半	差了很多
❑ +3	❑ +2	❑ +1	❑ 0	❑ -1	❑ -2	❑ -3

您的身心健康狀況

這項調查是詢問您對自己健康狀況的了解。此項資料記錄您的自我感覺和日常生活的情況。*謝謝您回答這份問卷！*

敬請回答下列各問題並在最適當的答案畫一個(☒)。

1. 總括來說，您認為您的健康狀況是：

極好	很好	好	一般	差
☐ 1	☐ 2	☐ 3	☐ 4	☐ 5

2. 下列問題是關於您日常生活中可能進行的活動。以您目前的健康狀況，您在進行這些活動時，有沒有受到限制？如果有的話，程度如何？

	有很大限制	有一點限制	沒有任何限制
a 中等強度的活動，比如搬桌子、使用吸塵器清潔地面、玩保齡球或打太極拳	☐ 1	☐ 2	☐ 3
b 上幾層樓梯	☐ 1	☐ 2	☐ 3

SF-12v2™ Health Survey © 1995, 2004 Health Assessment Lab, Medical Outcomes Trust and QualityMetric Incorporated. All rights reserved.
SF-12® is a registered trademark of Medical Outcomes Trust.
(IQOLA SF-12v2 Standard, Hong Kong (Chinese))

3. 在過去四個星期裏，您在工作或其它日常活動中，有多少時間會因為身體健康的原因而遇到下列的問題？

	常常如此	大部分時間	有時	偶爾	從來沒有
a 實際做完的比想做的要少	☐ 1	☐ 2	☐ 3	☐ 4	☐ 5
b 工作或其它活動的種類受到限制	☐ 1	☐ 2	☐ 3	☐ 4	☐ 5

4. 在過去的四個星期裏，您在工作或其它日常活動中，有多少時間由於情緒方面的原因（比如感到沮喪或焦慮）遇到下列的問題？

	常常如此	大部分時間	有時	偶爾	從來沒有
a 實際做完的比想做的要少	☐ 1	☐ 2	☐ 3	☐ 4	☐ 5
b 工作時或從事其它活動時不如往常細心了	☐ 1	☐ 2	☐ 3	☐ 4	☐ 5

5. 在過去四個星期裏，您身體上的疼痛對您的日常工作（包括上班和家務）有多大影響？

毫無影響	有很少影響	有一些影響	有較大影響	有極大影響
☐ 1	☐ 2	☐ 3	☐ 4	☐ 5

SF-12v2™ Health Survey © 1995, 2004 Health Assessment Lab, Medical Outcomes Trust and QualityMetric Incorporated. All rights reserved.
SF-12® is a registered trademark of Medical Outcomes Trust.
(IQOLA SF-12v2 Standard, Hong Kong (Chinese))

6. 下列問題是有關您在<u>過去四個星期裏</u>您覺得怎樣和您其它的情況。針對每一個問題，請選擇一個最接近您的感覺的答案。<u>在過去四個星期裏</u>，有多少時間：

	常常如此	大部分時間	有時	偶爾	從來沒有
a 您感到心平氣和？	☐ 1	☐ 2	☐ 3	☐ 4	☐ 5
b 您感到精力充足？	☐ 1	☐ 2	☐ 3	☐ 4	☐ 5
c 您覺得心情不好，悶悶不樂？	☐ 1	☐ 2	☐ 3	☐ 4	☐ 5

7. <u>在過去四個星期裏</u>，有多少時間由於您的<u>身體健康或情緒問題</u>妨礙了您的社交活動（比如探親、訪友等）？

常常有妨礙	大部分時間有妨礙	有時有妨礙	偶爾有妨礙	從來沒有妨礙
☐ 1	☐ 2	☐ 3	☐ 4	☐ 5

<u>2 星期</u>

<u>或六個月后我們會再通</u>過電話訪問來跟進您的健康情況（大約 30 分鐘）。

1. 願意參加：
   姓名:
   聯絡電話:
2. 不願意
3. 未能決定, 請在________________________再來電

－初診評估完－

SF-12v2™ Health Survey © 1995, 2004 Health Assessment Lab, Medical Outcomes Trust and QualityMetric Incorporated. All rights reserved.
SF-12® is a registered trademark of Medical Outcomes Trust.
(IQOLA SF-12v2 Standard, Hong Kong (Chinese))

Patient Follow up assessment form

醫院管理局中醫藥部
香港大學家庭醫學部

《測試中文 (香港版) 的中醫體質量表》

病人覆診評估量表

參加者姓名：________________________

參加日期：________________________

覆診日期：________________________

電話號碼：________________________

訪問員姓名：________________________

訪問員編號：________________________

SF-12v2™ Health Survey © 1995, 2004 Health Assessment Lab, Medical Outcomes Trust and QualityMetric Incorporated. All rights reserved.
SF-12® is a registered trademark of Medical Outcomes Trust.
(IQOLA SF-12v2 Standard, Hong Kong (Chinese))

 參加者編號：________

1. 請問你在過去兩個星期裏/六個月內有沒有再到診所求診?
   1. 沒有
   2. 有, a) _____次
      b) 為何症狀? ____________________________

2. 請問你在過去兩個星期裏/六個月內有沒有向其他醫生求診以治療你這個症狀?
   1. 沒有
   2. 有, 向其他西醫求診
   3. 有, 但我曾向其他中醫求診

3. 請問你在過去兩個星期裏/六個月內，除了服用這西醫診所的藥方，你有沒有服用其他藥物以治療你這個症狀?
   1. 沒有
   2. 有，中藥, 請註明：________________
   3. 有，西藥, 請註明：________________

整體病情改變評估

1. 跟你兩個星期前/六個月前到這診所的時候相比，你認為你的病情（情況）有改變嗎？

好了很多<br>❑ +2	好了一點<br>❑ +1	沒有轉變<br>❑ 0	差了一點<br>❑-1	差了很多<br>❑-2

SF-12v2™ Health Survey © 1995, 2004 Health Assessment Lab, Medical Outcomes Trust and QualityMetric Incorporated. All rights reserved.
SF-12® is a registered trademark of Medical Outcomes Trust.
(IQOLA SF-12v2 Standard, Hong Kong (Chinese))

 參加者編號：________

覆診(2 週/6 個月)

CCMQ 中醫體質量表（香港版）

本問卷是為了調查與你的體質有關的一些情況，從而為今後你的健康管理和臨床診治等提供參考。請閱讀每條問題，根據自己最近一年的實際情況或感覺，圈出最符合你的答案。如果你未能肯定如何回答某條問題時，請選擇最接近你實際情況的答案。

請注意：所有問題都是根據你最近一年的情況作答，而且每條問題只能選擇一個答案。

請根據最近一年的身體狀況和感覺，回答以下問題。	沒有(或不會)	很少	有時	大部份時間	常常
(1) 你精力充沛嗎？	1	2	3	4	5
(2) 你容易疲倦嗎？	1	2	3	4	5
(3) 你容易感到不够氣 (呼吸急促， 上氣不接下氣) 嗎？	1	2	3	4	5
(4) 你容易心慌嗎？	1	2	3	4	5
(5) 你容易頭暈或站起時暈眩嗎？	1	2	3	4	5
(6) 你喜歡安靜、懶得說話嗎?	1	2	3	4	5
(7) 你說話的聲音虛弱無力嗎?	1	2	3	4	5
(8) 你感到悶悶不樂、情緒低落嗎?	1	2	3	4	5

SF-12v2™ Health Survey © 1995, 2004 Health Assessment Lab, Medical Outcomes Trust and QualityMetric Incorporated. All rights reserved.
SF-12® is a registered trademark of Medical Outcomes Trust.
(IQOLA SF-12v2 Standard, Hong Kong (Chinese))

 參加者編號：________

<u>請根據最近一年的體驗和感覺，回答以下問題。</u>	**沒有 (或不會)**	**很少**	**有時**	**經常**	**常常**
(9) 你容易精神緊張、焦慮不安嗎?	1	2	3	4	5
(10) 你多愁善感、容易感到悲傷嗎？	1	2	3	4	5
(11) 你容易感到害怕或受驚嗎?	1	2	3	4	5
(12) 你肋骨部位或乳房脹痛嗎?	1	2	3	4	5
(13) 你感到胸悶或腹部脹滿嗎？	1	2	3	4	5
(14) 你會無緣無故歎氣嗎?	1	2	3	4	5
(15) 你感到身體沉重不輕鬆嗎?	1	2	3	4	5
(16) 你感到手心或腳掌心發熱嗎?	1	2	3	4	5
(17) 你手腳凍嗎?	1	2	3	4	5
(18) 你胃部、背部或腰膝部位怕冷嗎？	1	2	3	4	5
(19) 你怕冷、衣服**<u>較其他人</u>**穿得多嗎?	1	2	3	4	5
(20) **你沒有生病的情況下,** 你會感到身體、臉上發熱嗎？	1	2	3	4	5
(21) 你**<u>比一般人</u>**較不能抵受寒冷(如冬天的寒冷，夏天的空調冷氣、電扇等)嗎？	1	2	3	4	5
(22) 你**<u>比其他人</u>**較容易感冒嗎?	1	2	3	4	5

SF-12v2™ Health Survey © 1995, 2004 Health Assessment Lab, Medical Outcomes Trust and QualityMetric Incorporated. All rights reserved.
SF-12® is a registered trademark of Medical Outcomes Trust.
(IQOLA SF-12v2 Standard, Hong Kong (Chinese))

 參加者編號：________

請根據最近一年的體驗和感覺，回答以下問題。	**沒有 (或不會)**	**很少**	**有時**	**經常**	**常常**
(23) 你沒有感冒時也會打噴嚏嗎？	1	2	3	4	5
(24) 你沒有感冒時也會鼻塞、流鼻涕嗎?	1	2	3	4	5
(25) 你有因季節變化、溫度變化或異味等原因而咳嗽或喘氣的現象嗎？	1	2	3	4	5
(26) 你活動量稍大就容易出虛汗嗎? (虛汗的意思是指比一般人容易大量出汗)	1	2	3	4	5
(27) 你容易忘記事情（健忘）嗎？	1	2	3	4	5
(28) 你有額頭油脂分泌多的現象嗎?	1	2	3	4	5
(29) 你口唇的顏色比一般人較紅嗎?	1	2	3	4	5
(30) 你容易過敏(對藥物、食物、氣味、花粉或在季節交替、氣候變化時)嗎?	1	2	3	4	5
(31) 你的皮膚容易起風疹(包括風團、風疹塊、風疙瘩)嗎?	1	2	3	4	5
(32) 你的皮膚因過敏出現紫紅斑(即紫紅色瘀點、瘀斑)嗎?	1	2	3	4	5
(33) 你的皮膚在不知不覺中會出現青紫瘀斑(皮下出血)嗎?	1	2	3	4	5
(34) 你的皮膚一抓就紅，並出現抓痕嗎?	1	2	3	4	5
(35) 你皮膚或口唇乾嗎?	1	2	3	4	5
(36) 你面上顴骨兩面有細微紅絲嗎?	1	2	3	4	5

SF-12v2™ Health Survey © 1995, 2004 Health Assessment Lab, Medical Outcomes Trust and QualityMetric Incorporated. All rights reserved.
SF-12® is a registered trademark of Medical Outcomes Trust.
(IQOLA SF-12v2 Standard, Hong Kong (Chinese))

 參加者編號：________

請根據最近一年的體驗和感覺，回答以下問題。	**沒有 (或不會)**	**很少**	**有時**	**經常**	**常常**
(37) 你身體上有哪裡疼痛嗎？	1	2	3	4	5
(38) 你面部**兩顴**泛紅或偏紅嗎?	1	2	3	4	5
(39) 你面部或鼻部有油膩感或者油亮發光嗎?	1	2	3	4	5
(40) 你面色暗淡、或容易出現灰褐色斑嗎?	1	2	3	4	5
(41) 你易生粉刺、暗瘡或膿瘡嗎?	1	2	3	4	5
(42) 你上眼瞼**比別人**較腫（上眼瞼有輕微隆起的現象）嗎?	1	2	3	4	5
(43) 你容易有黑眼圈嗎?	1	2	3	4	5
(44) 你感到眼睛乾澀嗎？	1	2	3	4	5
(45) 你口唇顏色偏暗嗎?	1	2	3	4	5
(46) 你感到口乾喉燥、總想喝水嗎？	1	2	3	4	5
(47) 你覺得喉嚨部位有異物感，而且吐不出來，也無法吞下嗎？	1	2	3	4	5
(48) 你感到口苦或口腔有異味嗎?	1	2	3	4	5
(49) 你口腔有黏黏的感覺嗎?	1	2	3	4	5
(50) 你腹部肥滿鬆軟嗎?	1	2	3	4	5

SF-12v2™ Health Survey © 1995, 2004 Health Assessment Lab, Medical Outcomes Trust and QualityMetric Incorporated. All rights reserved.
SF-12® is a registered trademark of Medical Outcomes Trust.
(IQOLA SF-12v2 Standard, Hong Kong (Chinese))

請根據最近一年的體驗和感覺，回答以下問題。	**沒有 (或不會)**	**很少**	**有時**	**經常**	**常常**
(51) 你平時痰多，特別是喉嚨部位總感到有痰堵塞著嗎?	1	2	3	4	5
(52) 你**吃(喝)涼的食物**時會感到不舒服或者怕吃(喝)涼的食物嗎？	1	2	3	4	5
(53) 你能適應自然和社會環境的外在變化嗎？	1	2	3	4	5
(54) 你容易失眠嗎？	1	2	3	4	5
(55) 你**著涼或吃(喝)涼的食物後**，容易腹瀉嗎?	1	2	3	4	5
(56) 你的大便黏糊不爽、有未排清的感覺嗎?	1	2	3	4	5
(57) 你容易便秘或大便乾燥嗎?	1	2	3	4	5
(58) 你舌苔厚膩或有舌苔厚厚的感覺嗎?	1	2	3	4	5
(59) 你小便時尿道發熱、尿色深黃嗎？	1	2	3	4	5
(60) 你的「白帶」顏色偏黃嗎? **（限女性回答）**	1	2	3	4	5
(60) 你的陰囊部位潮濕嗎? **（限男性回答）**	1	2	3	4	5

SF-12v2™ Health Survey © 1995, 2004 Health Assessment Lab, Medical Outcomes Trust and QualityMetric Incorporated. All rights reserved.
SF-12® is a registered trademark of Medical Outcomes Trust.
(IQOLA SF-12v2 Standard, Hong Kong (Chinese))

A-7. 跟您三個月前相比，您認為您的健康情況有多少改變？

好了很多	好了一半	好了一點	沒有轉變	差了一點	差了一半	差了很多
❑ +3	❑ +2	❑ +1	❑ 0	❑ -1	❑ -2	❑ -3

您的身心健康狀況

這項調查是詢問您對自己健康狀況的了解。此項資料記錄您的自我感覺和日常生活的情況。*謝謝您回答這份問卷！*

敬請回答下列各問題並在最適當的答案畫一個(☒)。

7. 總括來說，您認為您的健康狀況是：

極好	很好	好	一般	差
☐ 1	☐ 2	☐ 3	☐ 4	☐ 5

8. 下列問題是關於您日常生活中可能進行的活動。以您目前的健康狀況，您在進行這些活動時，有沒有受到限制？如果有的話，程度如何？

		有很大限制	有一點限制	沒有任何限制
a	中等強度的活動，比如搬桌子、使用吸塵器清潔地面、玩保齡球或打太極拳	☐ 1	☐ 2	☐ 3
b	上幾層樓梯	☐ 1	☐ 2	☐ 3

SF-12v2™ Health Survey © 1995, 2004 Health Assessment Lab, Medical Outcomes Trust and QualityMetric Incorporated. All rights reserved.
SF-12® is a registered trademark of Medical Outcomes Trust.
(IQOLA SF-12v2 Standard, Hong Kong (Chinese))

9. 在過去四個星期裏，您在工作或其它日常活動中，有多少時間會因為身體健康的原因而遇到下列的問題？

	常常如此	大部分時間	有時	偶爾	從來沒有
a 實際做完的比想做的要少	☐ 1	☐ 2	☐ 3	☐ 4	☐ 5
b 工作或其它活動的種類受到限制	☐ 1	☐ 2	☐ 3	☐ 4	☐ 5

10. 在過去的四個星期裏，您在工作或其它日常活動中，有多少時間由於情緒方面的原因（比如感到沮喪或焦慮）遇到下列的問題？

	常常如此	大部分時間	有時	偶爾	從來沒有
a 實際做完的比想做的要少	☐ 1	☐ 2	☐ 3	☐ 4	☐ 5
b 工作時或從事其它活動時不如往常細心了	☐ 1	☐ 2	☐ 3	☐ 4	☐ 5

11. 在過去四個星期裏，您身體上的疼痛對您的日常工作（包括上班和家務）有多大影響？

毫無影響	有很少影響	有一些影響	有較大影響	有極大影響
☐ 1	☐ 2	☐ 3	☐ 4	☐ 5

SF-12v2™ Health Survey © 1995, 2004 Health Assessment Lab, Medical Outcomes Trust and QualityMetric Incorporated. All rights reserved.
SF-12® is a registered trademark of Medical Outcomes Trust.
(IQOLA SF-12v2 Standard, Hong Kong (Chinese))

12. **下列問題是有關您在<u>過去四個星期裏</u>您覺得怎樣和您其它的情況。針對每一個問題，請選擇一個最接近您的感覺的答案。<u>在過去四個星期裏</u>，有多少時間：**

		常常如此	大部分時間	有時	偶爾	從來沒有
a	您感到心平氣和？	☐ 1	☐ 2	☐ 3	☐ 4	☐ 5
b	您感到精力充足？	☐ 1	☐ 2	☐ 3	☐ 4	☐ 5
c	您覺得心情不好，悶悶不樂？	☐ 1	☐ 2	☐ 3	☐ 4	☐ 5

7. **<u>在過去四個星期裏</u>，有多少時間由於您的<u>身體健康或情緒問題</u>妨礙了您的社交活動（比如探親、訪友等）？**

常常有妨礙	大部分時間有妨礙	有時有妨礙	偶爾有妨礙	從來沒有妨礙
☐ 1	☐ 2	☐ 3	☐ 4	☐ 5

問卷完成，多謝你的幫助！

<u>六個月后</u>我們會再通過電話訪問來跟進您的健康情況（大約 30 分鐘）。

2. 願意參加：
2. 不願意
3. 未能決定, 請在________________________再來電

SF-12v2™ Health Survey © 1995, 2004 Health Assessment Lab, Medical Outcomes Trust and QualityMetric Incorporated. All rights reserved.
SF-12® is a registered trademark of Medical Outcomes Trust.
(IQOLA SF-12v2 Standard, Hong Kong (Chinese))

Appendix C

醫院管理局中醫藥部及香港大學家庭醫學部
測試中文 (香港版) 的中醫體質量表(CCMQ)

參加者編號：________

中醫師評估

1. 請顯示以下 3 種體質，那種最能代表病人的情況及其嚴重情度 (由中醫師填寫)

	**a)主要體質**	**b)伴隨體質**	**(c) 嚴重程度**				
**體質 I**	**(只選一項)**	**(只選一項)**	極輕微	輕微	中度	嚴重	極嚴重
陽虛型	☐(1)	☐(1)	☐(1)	☐(2)	☐(3)	☐(4)	☐(5)
陰虛型	☐(2)	☐(2)	☐(1)	☐(2)	☐(3)	☐(4)	☐(5)
痰瘀型	☐(3)	☐(3)	☐(1)	☐(2)	☐(3)	☐(4)	☐(5)
平和型	☐(5)	☐(6)	☐(1)	☐(2)	☐(3)	☐(4)	☐(5)
不能分辨	☐(4)	☐(4)	☐(1)	☐(2)	☐(3)	☐(4)	☐(5)
Office use		NA (5)	(c) if NA(6)				

2. 請評估病人所患的疾病的嚴重程度。(由中醫師填寫)

極輕微	輕微	中度	嚴重	極嚴重
☐ (1)	☐ (2)	☐ (3)	☐ (4)	☐ (5)

3. 另外，請顯示以下 9 種體質，最能代表病人的情況及其嚴重程度 (由中醫師填寫)

	**a)主要體質**	**b)伴隨體質**	**(c) 嚴重程度**				
**體質 II**	**(只選一項)**	**(只選一項)**	極輕微	輕微	中度	嚴重	極嚴重
平和型	☐(1)	☐(1)	☐(1)	☐(2)	☐(3)	☐(4)	☐(5)
氣虛型	☐(2)	☐(2)	☐(1)	☐(2)	☐(3)	☐(4)	☐(5)
陽虛型	☐(3)	☐(3)	☐(1)	☐(2)	☐(3)	☐(4)	☐(5)
陰虛型	☐(4)	☐(4)	☐(1)	☐(2)	☐(3)	☐(4)	☐(5)
痰濕型	☐(5)	☐(5)	☐(1)	☐(2)	☐(3)	☐(4)	☐(5)
濕熱型	☐(6)	☐(6)	☐(1)	☐(2)	☐(3)	☐(4)	☐(5)
血瘀型	☐(7)	☐(7)	☐(1)	☐(2)	☐(3)	☐(4)	☐(5)
氣鬱型	☐(8)	☐(8)	☐(1)	☐(2)	☐(3)	☐(4)	☐(5)
特稟型	☐(9)	☐(9)	☐(1)	☐(2)	☐(3)	☐(4)	☐(5)
不能分辨	☐(10)	☐(10)	☐(1)	☐(2)	☐(3)	☐(4)	☐(5)
Office use		NA (11)	(c) if NA(6)				

中醫師姓名(請用正楷寫上)： ________________________

簽署： ________________________

中醫體質量表（香港版）

Constitution in Chinese Medicine Questionnaire, CCMQ (HK Version)

「中醫體質量表（Constitution in Chinese Medicine Questionnaire, CCMQ）」係由北京中國醫藥大學中醫學院王琦教授領導之體質與證型研究團隊所開發完成，於2006年完成第一版問卷。

版權所有 翻印必究

CCMQ_60 China Standard Version 1.0

Copyright © 2006 Chinese Medicine Constitution Research Centre. All rights reserved.

Appendix D

本問卷是為了調查與你的體質有關的一些情況，從而為今後你的健康管理和臨床診治等提供參考。請閱讀每條問題，根據自己最近一年的實際情況或感覺，圈出最符合你的答案。如果你未能肯定如何回答某條問題時，請選擇最接近你實際情況的答案。

請注意：所有問題都是根據你最近一年的情況作答，而且每條問題只能選擇一個答案。

請根據最近一年的身體狀況和感覺，回答以下問題。	沒有(或不會)	很少	有時	大部份時間	常常
(1) 你精力充沛嗎？	1	2	3	4	5
(2) 你容易疲倦嗎？	1	2	3	4	5
(3) 你容易感到不够氣 (呼吸急促， 上氣不接下氣) 嗎？	1	2	3	4	5
(4) 你容易心慌嗎？	1	2	3	4	5
(5) 你容易頭暈或站起時暈眩嗎？	1	2	3	4	5
(6) 你喜歡安靜、懶得說話嗎?	1	2	3	4	5
(7) 你說話的聲音虛弱無力嗎?	1	2	3	4	5
(8) 你感到悶悶不樂、情緒低落嗎?	1	2	3	4	5

請根據最近一年的體驗和感覺，回答以下問題。	**沒有(或不會)**	**很少**	**有時**	**經常**	**常常**
(9) 你容易精神緊張、焦慮不安嗎?	1	2	3	4	5
(10) 你多愁善感、容易感到悲傷嗎？	1	2	3	4	5

(11) 你容易感到害怕或受驚嗎?	1	2	3	4	5
(12) 你肋骨部位或乳房脹痛嗎?	1	2	3	4	5
(13) 你感到胸悶或腹部脹滿嗎？	1	2	3	4	5
(14) 你會無緣無故歎氣嗎?	1	2	3	4	5
(15) 你感到身體沉重不輕鬆嗎?	1	2	3	4	5
(16) 你感到手心或腳掌心發熱嗎?	1	2	3	4	5
(17) 你手腳凍嗎?	1	2	3	4	5
(18) 你胃部、背部或腰膝部位怕冷嗎？	1	2	3	4	5
(19) 你怕冷、衣服**較其他人**穿得多嗎?	1	2	3	4	5
(20) **你沒有生病的情況下,** 你會感到身體、臉上發熱嗎？	1	2	3	4	5
(21) 你**比一般人**較不能抵受寒冷(如冬天的寒冷，夏天的空調冷氣、電扇等)嗎？	1	2	3	4	5
(22) 你**比其他人**較容易感冒嗎?	1	2	3	4	5

請根據最近一年的體驗和感覺，回答以下問題。	**沒有 (或不會)**	**很少**	**有時**	**經常**	**常常**
(23) 你**沒有感冒時**也會打噴嚏嗎？	1	2	3	4	5

(24) 你沒有感冒時也會鼻塞、流鼻涕嗎?	1	2	3	4	5
(25) 你有因季節變化、溫度變化或異味等原因而咳嗽或喘氣的現象嗎？	1	2	3	4	5
(26) 你活動量稍大就容易出虛汗嗎?<br>(虛汗的意思是指比一般人容易大量出汗)	1	2	3	4	5
(27) 你容易忘記事情（健忘）嗎？	1	2	3	4	5
(28) 你有額頭油脂分泌多的現象嗎?	1	2	3	4	5
(29) 你口唇的顏色比一般人較紅嗎?	1	2	3	4	5
(30) 你容易過敏(對藥物、食物、氣味、花粉或在季節交替、氣候變化時)嗎?	1	2	3	4	5
(31) 你的皮膚容易起風疹(包括風團、風疹塊、風疙瘩)嗎?	1	2	3	4	5
(32) 你的皮膚因過敏出現紫紅斑(即紫紅色瘀點、瘀斑)嗎?	1	2	3	4	5
(33) 你的皮膚在不知不覺中會出現青紫瘀斑(皮下出血)嗎?	1	2	3	4	5
(34) 你的皮膚一抓就紅，並出現抓痕嗎?	1	2	3	4	5
(35) 你皮膚或口唇乾嗎?	1	2	3	4	5
(36) 你面上顴骨兩面有細微紅絲嗎?	1	2	3	4	5
請根據最近一年的體驗和感覺，回答以下問題。	**沒有<br>(或不會)**	**很少**	**有時**	**經常**	**常常**
(37) 你身體上有哪裡疼痛嗎？	1	2	3	4	5

(38) 你面部**兩顴**泛紅或偏紅嗎?	1	2	3	4	5
(39) 你面部或鼻部有油膩感或者油亮發光嗎?	1	2	3	4	5
(40) 你面色暗淡、或容易出現灰褐色斑嗎?	1	2	3	4	5
(41) 你易生粉刺、暗瘡或膿瘡嗎?	1	2	3	4	5
(42) 你上眼瞼**比別人**較腫（上眼瞼有輕微隆起的現象）嗎?	1	2	3	4	5
(43) 你容易有黑眼圈嗎?	1	2	3	4	5
(44) 你感到眼睛乾澀嗎？	1	2	3	4	5
(45) 你口唇顏色偏暗嗎?	1	2	3	4	5
(46) 你感到口乾喉燥、總想喝水嗎？	1	2	3	4	5
(47) 你覺得喉嚨部位有異物感，而且吐不出來，也無法吞下嗎？	1	2	3	4	5
(48) 你感到口苦或口腔有異味嗎?	1	2	3	4	5
(49) 你口腔有黏黏的感覺嗎?	1	2	3	4	5
(50) 你腹部肥滿鬆軟嗎?	1	2	3	4	5
請根據最近一年的體驗和感覺，回答以下問題。	**沒有 (或不會)**	**很少**	**有時**	**經常**	**常常**
(51) 你平時痰多，特別是喉嚨部位總感到有痰堵塞著嗎?	1	2	3	4	5

(52) 你**<u>吃(喝)涼的食物</u>**時會感到不舒服或者怕吃(喝)涼的食物嗎？	1	2	3	4	5
(53) 你能適應自然和社會環境的外在變化嗎？	1	2	3	4	5
(54) 你容易失眠嗎？	1	2	3	4	5
(55) 你**<u>著涼或吃(喝)涼的食物後</u>**，容易腹瀉嗎?	1	2	3	4	5
(56) 你的大便黏糊不爽、有未排清的感覺嗎?	1	2	3	4	5
(57) 你容易便秘或大便乾燥嗎?	1	2	3	4	5
(58) 你舌苔厚膩或有舌苔厚厚的感覺嗎?	1	2	3	4	5
(59) 你小便時尿道發熱、尿色深黃嗎？	1	2	3	4	5
(60) 你的「白帶」顏色偏黃嗎?（**限女性回答**）	1	2	3	4	5
(60) 你的陰囊部位潮濕嗎?（**限男性回答**）	1	2	3	4	5
